# Molecular, histopathological, and clinical risk factors for overall metastatic disease and brain metastases in surgically resected stage I–III lung cancer

**DOI:** 10.1007/s10585-026-10444-8

**Published:** 2026-10-23

**Authors:** Anne Pernille Harlem Dyrbekk, Pål Suhrke, Marianne Odnakk Ludahl, Nermin Zecic, Joakim Oliu Moe, Inger Johanne Zwicky Eide, Marius Lund-Iversen, Odd Terje Brustugun

**Affiliations:** 1 University of Oslo, Oslo, 0316 Norway; 2 Department of Pathology, Vestfold Hospital Trust, Tønsberg, 3103 Norway; 3 Department of Cancer Genetics, Institute for Cancer Research, The Norwegian Radium Hospital, Oslo, 0310 Norway; 4 Department of Pathology, Oslo University Hospital, The Norwegian Radium Hospital, Oslo, 0310 Norway; 5 Department of Microbiology, Division for gene-technology, Vestfold Hospital Trust, Tønsberg, 3103 Norway; 6 Department of Gastroenterology, Vestfold Hospital Trust, Tønsberg, 3103 Norway; 7 Department of Oncology, Vestre Viken Hospital Trust, Drammen, 3004 Norway

**Keywords:** Brain metastasis, Lung cancer, Risk factors, Next-generation sequencing

## Abstract

Lung cancer remains a major cause of cancer-related mortality, including among patients diagnosed with potentially curable disease. Current decisions regarding perioperative treatment and follow-up rely on a limited number of established prognostic factors. Further knowledge is needed regarding the clinical, histopathological, and molecular factors associated with subsequent metastatic disease, particularly brain metastasis (BM), which is associated with poor prognosis. We investigated these factors in a large cohort of patients with surgically resected stage I–III lung cancer followed for more than five years. BM was associated with higher pathological tumour and lymph-node stage and younger age at surgery. Lung adenocarcinoma was associated with a higher risk of BM than lung squamous cell carcinoma. Several molecular alterations were associated with metastatic disease or BM, including *KRAS* mutations in lung adenocarcinoma and *NFE2L2* mutations in lung squamous cell carcinoma. Additional associations involving specific *EGFR* and *KRAS* mutation subtypes and rarer alterations were observed, but were based on small numbers of cases and should be considered exploratory. Several of the associated genes are involved in the RAS/MAPK, PI3K/AKT/mTOR, or NRF2-related pathways. However, pathway activation and causality could not be established in this observational study. These findings support further investigation of clinical, histopathological, and molecular factors associated with metastatic progression after surgical resection. Validation in larger, independent, stage-stratified and contemporary cohorts with comprehensive treatment data is required before the identified molecular alterations can be considered for clinical risk stratification or surveillance.

**Supplementary Information:**

The online version contains supplementary material available at https://doi.org/10.1007/s10585-026-10444-8.

## Introduction

Lung cancer is one of the most prevalent types of cancer worldwide and remains a leading cause of cancer-related mortality both in Norway and worldwide [1, 2]. At diagnosis, approximately 42% of patients present with advanced stage IV disease, whereas 55% are diagnosed at potentially curable stages I–III [3]. Despite achieving R0 complete resection, 30–50% of surgically treated patients experience relapse, often reflecting disseminated disease and poor prognosis, particularly in patients who develop brain metastasis (BM) [4, 5].

Advances in translational research have provided important insight into the molecular mechanisms underlying tumourigenesis and metastatic propensity [6]. This has enabled the development of targeted therapies and substantially altered treatment strategies across several malignancies. For example, mutations and gene fusions involving proto-oncogenes such as *EGFR* and *ALK* result in constitutive kinase activation and uncontrolled cell proliferation. Targeted therapies directed against these alterations have significantly improved survival in advanced lung cancer patients [7, 8]. More recently, such therapies have also been incorporated into adjuvant treatment regimens for early-stage disease [9]. These developments illustrate a broader shift in oncology from treatment strategies on the basis primarily of the tumour site of origin and anatomical stage towards approaches increasingly guided by tumour biology. Molecular characteristics may provide prognostic and predictive information beyond conventional staging and could improve patient selection for treatment and follow-up.

Adjuvant therapy is offered to selected lung cancer patients following surgical resection to reduce the risk of recurrence and metastasis. Although treatment decisions incorporate clinical and molecular factors, established prognostic markers for guiding adjuvant therapy remain limited. Currently, decision-making relies mainly on histopathological parameters contributing to TNM staging, including tumour size, local invasion, and lymph node involvement [10]. According to updated ESMO guidelines, selected subgroups may also be considered for neoadjuvant or adjuvant therapy on the basis of the status of molecular markers such as PD-L1, *ALK*, and *EGFR*. Postoperatively, patients are followed with computed tomography (CT) every six months for two years and annually thereafter for three additional years. Magnetic resonance imaging (MRI) of the brain is typically reserved for patients presenting with neurological symptoms [9]. Consequently, asymptomatic BM may remain undetected until neurological symptoms develop, highlighting the need for improved risk stratification strategies.

Despite advances in molecular profiling, the molecular determinants of metastasis in lung cancer remain poorly understood. Several studies have explored potential biomarkers, including those associated with BM. Garinet et al. [4] provided a comprehensive overview of prognostic factors in early-stage lung cancer, highlighting molecular alterations at the DNA, RNA, and epigenetic levels. Huang et al. analysed molecular profiles from 3035 non-small cell lung cancer (NSCLC) BM cases and 7277 primary NSCLC tumours using routine next-generation sequencing (NGS). They reported a higher prevalence of targetable alterations in BMs, including *ALK* fusions, *KRAS* G12C mutations, and *MET* amplifications, alongside reduced *MET* exon 14 skipping. Furthermore, paired analyses demonstrated that 85% of BMs harboured at least one additional genetic alteration compared with the corresponding primary tumour [11]. These findings suggest that molecular evolution may occur during metastatic progression and underscore the value of comparing paired primary and metastatic lesions.

Improved identification of biological predictors of metastasis could enhance risk stratification, guide (neo)adjuvant treatment decisions, and optimize follow-up strategies. In this study, we investigated the clinical, histopathological, and molecular risk factors for metastasis in a large cohort of patients with surgically resected stage I–III lung cancer. Given the particularly poor prognosis associated with BM, we place special emphasis on this outcome. We also compare genetic alterations between primary and metastatic tumours to improve our understanding of metastatic progression. We hypothesize that previously reported findings will be reproducible in this Norwegian cohort and that additional potential biomarkers associated with metastatic progression may be identified.

## Methods

### Patients

This study employed data and specimens from a cohort of patients with lung cancer who underwent surgical resection at Oslo University Hospital (OUH) in Norway between 2006 and 2018. The cohort is supported by a biobank containing fresh-frozen resected tumour tissue and a database with relevant histopathological information and clinical data, including long-term follow-up data. The database includes various predictive parameters, including the results of *EGFR* and *KRAS* analyses performed as part of routine diagnostics at OUH.

Histopathological diagnoses were initially established as part of routine diagnostics at the time of diagnosis. However, substantial changes occurred in the WHO Classification of Tumours of the Lung, Pleura, Thymus and Heart between 2004 and 2015 editions, particularly following the incorporation of immunohistochemistry into the diagnostic algorithm [12, 13]. To ensure diagnostic consistency across the study period, all tumours were subsequently reviewed and reclassified according to the 2015 classification [12]. Histopathological classification was based on an integrated assessment of morphological and immunohistochemical findings. In most cases, immunohistochemical evaluation included TTF-1 and p40 to support subtype classification, while neuroendocrine markers were applied when appropriate. The majority of the cases were also assessed for NUT protein expression, and no NUT carcinomas were identified [14]. Cases diagnosed as large cell neuroendocrine carcinoma (LCNEC) underwent additional histopathological review and were included in a collaborative study with a reference center in Lyon [15].

Patient follow-up was conducted by a dedicated study nurse who systematically collected data from medical records at OUH as well as from local hospitals. Follow-up continued until May 1, 2021. Data on all-cause and lung cancer-specific deaths were obtained through direct linkage to the Norwegian Cause of Death Registry and validated using information from electronic medical records [16].

Typically, metastases were diagnosed through routine radiological assessments using CT scans. Magnetic resonance imaging (MRI) of the brain was used when there was clinical suspicion of BM. Patients with carcinoid tumours and those with stage IV disease at the time of surgery were excluded. None of the patients received neoadjuvant treatment. During the study period, patients with stage II–III NSCLC who were younger than 70 years and had an Eastern Cooperative Oncology Group (ECOG) performance status of 0–1 were offered adjuvant platinum-doublet chemotherapy (cisplatin combined with vinorelbine) for up to four cycles according to treatment guidelines. However, complete information on actual treatment received was not available for all patients. Likewise, information about treatment after progression was not available. Therefore, treatment exposure was not included as a covariate in the analyses. All participants provided written informed consent, and the Regional Ethics Committee approved the project (number 2009/1904, S-06402b).

The database includes NGS results from primary tumours, primarly generated from previous studies of ROS1 and TRK protein expression [17, 18]. In the present study, we performed additional sequencing of all available metastatic tissue and paired sequencing of the corresponding primary tumours. Cases with available NGS data were selected on the basis of tissue availability and inclusion in previous molecular studies and were not selected according to metastatic outcome.

### Specimen characteristics

DNA and RNA were extracted from fresh-frozen tissue samples obtained from the resected specimens. Details regarding the extraction protocols are provided in the Online resource/Supplementary Information. The tissue samples and extracted nucleic acids were stored at − 80 °C. The tumour percentage was evaluated in frozen sections. For a small subset of samples with a tumour content below 10%, DNA and RNA were re-extracted from formalin-fixed paraffin-embedded (FFPE) tissue blocks, followed by repeat sequencing. For patients who underwent subsequent resection of metastatic lesions, sequencing was performed on FFPE tissue blocks from all available metastatic specimens.

### Assay methods: sequencing

We performed library preparation on an Ion Chef instrument (Thermo Fisher Scientific), followed by sequencing on the Ion Torrent S5/Ion Torrent S5 XL platforms and Ion Torrent server (Ubuntu v14.04-18.04 LTS) using the Oncomine Comprehensive Assay V3 panel (Thermo Fisher Scientific, A35806) at OUH or Vestfold Hospital Trust. The panel covers single nucleotide variants (SNVs), insertions and deletions (indels), copy number variations (CNVs), and gene fusions across 161 genes. Variant calling was performed using Ion Reporter software version 5.10–5.18 with the Oncomine Variants filter, applying a CNV threshold corresponding to a copy number gain of ≥ 2 relative to normal at a 5% confidence interval. For cases with paired sequencing of primary and metastatic tumours, the extended filter and the Karolinska Database/Molecular Tumor Board Portal were used to assess variant pathogenicity [19]. Only variants categorised as pathogenic or likely pathogenic were included in the analyses [20]. Additional details regarding these protocols are provided in the Online resource/Supplementary Information.

### Dependent and independent variables

The main dependent variables were the occurrence of metastatic disease, time to metastasis, early metastasis (< 6 months), lung cancer-specific death, all-cause death, and time from metastasis to death. Metastases were categorised as either BM or extracranial metastasis (ECM) according to the site of first radiologically detected metastatic occurrence, allowing separate analyses of these outcomes. Patients with BM, including those with synchronous brain and ECM at first presentation, were classified in the BM group. Patients with ECM without synchronous BM were classified in the ECM group. Since brain MRI was generally performed only when there was clinical suspicion of BM, the recorded timing of BM reflects the timing of radiological detection rather than the true biological onset of intracranial metastatic disease. Local recurrence without distant metastasis was not classified as metastatic disease. Early metastasis was defined as metastatic disease diagnosed within six months after surgery. This cut-off was selected because the first routine postoperative surveillance assessment after curative-intent surgery for NSCLC is scheduled at approximately six months according to Norwegian lung cancer guidelines and generally includes a clinical evaluation and CT imaging of the thorax and adrenal glands [10].

The independent variables were sex, age, disease stage, smoking history, histopathological diagnosis and NGS/polymerase chain reaction (PCR) results. Smoking history was categorised as former/current smoker or never smoker (< 100 cigarettes smoked during lifetime). Tobacco exposure was quantified in pack-years and was calculated as the average number of cigarette packs smoked per day multiplied by the number of years smoking.

### Study design and statistical analysis

This study was based on a prospectively established clinical cohort and biobank with linked registry data and with retrospective molecular analysis. The data were initially explored using frequency tables. Associations between independent and dependent variables were assessed using univariate or multivariate logistic regression analyses. For outcomes subject to censoring or variable follow-up time, survival analyses were performed using Kaplan-Meier estimates with log rank tests and univariable or multivariable Cox proportional hazards regression models. The proportional hazards assumption was assessed using Schoenfeld residuals.

Two-sided *p*-values ≤ 0.05 were considered statistically significant. Given the low prevalence of specific molecular alterations, associations with *p*-values ≤ 0.10 are also presented and discussed as borderline or exploratory findings. Given the exploratory nature of the statistical analyses, no formal correction for multiple testing was applied. Results should therefore be interpreted as hypothesis-generating.

For descriptive purposes, we calculated the median observed time to event among patients who experienced the event during follow-up. Patients who did not experience the event were excluded from these conditional summaries, but were included as censored observations in the Kaplan-Meier and Cox regression analyses.

STATA Release 18 was used for statistical analysis [21].

## Results

### Basic clinicopathological parameters and associations with later metastasis

The cohort included 912 patients with resected, stage I–III lung cancer (Table 1). Two patients were lost to follow-up (emigration) and were excluded. The median follow-up time was 5.42 years. A total of 51.5% were men, 48.5% were women, and the median age was 67.6 years (range 39.2–87.0). Most patients had a smoking history (93.1%), but 50.9% of the patients with a smoking history had quit smoking 1 year or more prior to diagnosis (Online resource A1). Only 7.0% had never smoked. The most common histopathological type was lung adenocarcinoma (LUAD) (58.4%), followed by lung squamous cell carcinoma (LUSC) (32.9%) and large cell carcinoma (LCC) (2.9%). The majority were in P-stages I and II (84.8%), T-stages 1a-2b (81.9%), and N-stage 0 (72.4%) **(**Table 1 and Online resource A2).

In total, 344 (37.7%) patients developed metastatic disease during the study period. Among patients who developed metastases, the median time to metastasis was 1.48 years, ranging from 0.53 years for undifferentiated carcinomas to 2.39 years for salivary gland carcinomas. Relative to LUSC, Cox regression and log-rank analyses revealed significantly increased metastatic risk in several histopathological subtypes, including mixed carcinoma, undifferentiated carcinoma, LCNEC, adenosquamous carcinoma (ADSQC), LCC and LUAD (Table 1 and Online resource A3). There were no significant associations between age, age groups (≥/<60 years), sex, smoking history (never smoking versus active/former smoking and active versus former smoking), and later metastasis in general (Table 1 and Online resource A1). There seemed to be an increasing gradient in metastasis risk with increasing P-stage, T-stage and N-stage (Table 1 and Online resource A2). N-stage was associated with a higher hazard ratio (HR) than T-stage was.

Among the 344 patients who developed metastatic disease, 32.8% had BM, and 67.2% had ECM as the first metastatic event. The median time to BM was 1.01 years. BM was more common in younger patients (especially those under 60 years) and those with fewer pack-years of smoking (Tables 1 and 3 and Online resource A3). Patients < 60 years had longer median follow-up time than patients ≥ 60 years (6.4 and 5.2 years, respectively). Among patients who developed BM, the median time from surgery to BM was 0.9 years in those < 60 years and 1.1 years in those ≥ 60 years. Compared with that of LUSC, the risk of BM was greater in LUAD, small cell carcinoma (SCLC), ADSQC, LCNEC and mixed carcinoma (few cases) (Table 1 and Online resource A3). The hazard of BM was 3.62 times greater in LUAD than in LUSC. For LUAD, the HR for BM increased with increasing P-stage. None of the patients with stage Ia or Ib LUSC (*n* = 153) developed BM during follow-up, and no association was observed between a more advanced stage and the risk of BM in LUSC, although the number of BM cases was low (Table 1, Online resource A3, A4, and A5). For information on the ECM, see Online resource A6. The Kaplan‒Meier plots are provided in Online resource Fig. A1a‒f.

Among the 344 patients with subsequent metastasis, 59 (17.2%) experienced early metastasis (< 6 months post-surgery), with a lower risk in those aged > 50 years at the time of surgery. Compared with LUSC, early metastasis was more common in LCC patients (OR 3.38, 95% CI 1.03–11.08, *p* = 0.045). The risk also increased with increasing pT, pN, and P-stages and male sex (Online resource A7). Patients who had BM as their first metastatic site had 1.8 times greater odds of developing metastases before 0.5 years than did those who had ECM as their first metastatic site (OR 1.80; 95% CI 1.01–3.18, *p* = 0.046).

The median time from surgery to all-cause death in the whole cohort was 2.94 years, and the median time to lung cancer death was 2.43 years. Among patients who developed BM, the median time to lung cancer death was 2.45 years (Online resource A8). After BM, the median time to death was 0.73 years. Patients with a smoking history showed a trend toward shorter survival after BM than patients without a smoking history (HR 2.04, 95% CI 0.98–4.23, *p* = 0.056) (Online resource A3). In LUAD, the initial P-stage did not seem to affect the time to death after BM or ECM (Online resource Table A5). The same pattern was observed for both all-cause death and lung cancer death.

**Table 1:** Demographics, histopathological characteristics, risk of metastasis and risk of brain metastasis

	All (% of total)	Metastatic*	Nonmetastatic	Risk of met. Total: all cases (*N* = 912) HR, 95% CI, *p*-value	BM	Risk of BM Total: all cases (*n* = 912) HR, 95% CI, *p*-value
*Number *	912 (100%)	344	568		113	
*Sex*						
Female	442 (48.5%)	167 (48.6%)	275 (48.4%)	Ref	60 (53.1%)	Ref
Male	470 (51.5%)	177 (51.5%)	293 (51.6%)	N.S.	53 (46.9%)	N.S.
* Age at surgery (median)*	67.6	67.3	67.8	N.S.	65.3	0.97, 0.9-1.00, *p* = 0.029
*Age group 60*						
< 60 years	171 (18.8%)	71 (20.6%)	100 (17.6%)	Ref	31 (27.4%)	Ref
≥ 60 years	741 (81.3%)	273 (79.4%)	468 (82.4%)	N.S.	82 (72.7%)	0.63, 0.42–0.95, *p* = 0.027
*Smoking*						
Never smoker	64 (7.0%)	24 (7.0%)	40 (7.0%)	Ref	10 (8.9%)	Ref
Former/current smoker	848 (93.1%)	320 (93.0%)	528 (93.0%)	N.S.	103 (91.2%)	N.S.
Pack year (median)	31.5	31.8	30.8	N.S.	26.6	0.99, 0.98-1.00, *p* = 0.033
*Histology*						
LUSC	300 (32.9%)	88 (25.6%)	212 (37.3%)	Ref	13 (11.5%)	Ref
LUAD	533 (58.4%)	212 (61.6%)	321 (56.5%)	1.33, 1.04–1.70, *p* = 0.025	85 (75.2%)	3.62, 2.02–6.50, *p* = < 0.001
LCC	26 (2.9%)	14 (4.1%)	12 (2.1%)	2.42, 1.38–4.26, *p* = 0.002	3 (2.7%)	3.46, 0.98–12.13, *p* = 0.053
ADSQC	16 (1.8%)	11 (3.1%)	5 (0.9%)	2.89, 1.54–5.41, *p* = 0.001	4 (3.5%)	6.71, 2.19–20.58, *p* = 0.001
SCLC	13 (1.4%)	6 (1.7%)	7 (1.2%)	N.S.	4 (3.5%)	6.88, 2.24–21.09, *p* = 0.001
LCNEC	10 (1.1%)	7 (2.0%)	3 (0.5%)	3.46, 1.60–7.47, *p* = 0.002	3 (2.7%)	8.85, 2.52–31.13, *p* = 0.001
Sal. gl.-tp. carc.	5 (0.6%)	2 (0.6%)	3 (0.5%)	n.s.	–	–
Undiff. carc.	4 (0.4%)	3 (0.9%)	1 (0.2%)	5.80, 1.83–18.38, *p* = 0.003	–	–
Other	3 (0.3%)	–	3 (0.5%)	–	–	–
Mixed	2 (0.2%)	1 (0.3%)	1 (0.2%)	12.88, 1.78–93.32, *p* = 0.011	1 (0.9%)	61.84, 7.94-481.57. *p* < 0.001
*P-stage*						
Ia	259 (28.4%)	63 (18.3%)	196 (34.5%)	Ref	16 (14.2%)	Ref
Ib	242 (26.5%)	84 (24.4%)	158 (27.8%)	1.56, 1.13–2.17, *p* = 0.007	24 (21.2%)	1.72, 0.92–3.24, *p* = 0.092
IIa	177 (19.4%)	76 (22.1%)	101 (17.8%)	2.20, 1.58–3.08, *p* < 0.001	24 (21.2%)	2.63, 1.40–4.95, *p* = 0.003
IIb	95 (10.4%)	46 (13.4%)	49 (8.6%)	2.65, 1.81–3.87, *p* < 0.001	14 (12.4%)	3.06, 1.49–6.27, *p* = 0.002
IIIa	134 (14.7%)	73 (21.2%)	61 (10.7%)	3.36, 2.40–4.72, p = < 0.001	34 (30.1%)	5.68, 3.13–10.30, *p* < 0.001
IIIb	3 (0.3%)	2 (0.6%)	1 (0.2%)	8.59, 2.10-35.17, *p* = 0.003	1 (0.9%)	14.39, 1.90-108.73, *p* = 0.010
Missing	2 (0.2%)	–	2 (0.4%)	–	–	

*Develops metastasis later. N.S.: Not significant (*p* > 0.1). HR: hazard ratio. 95% CI: 95% confidence interval. Ref: Reference group. BM: Brain metastasis LUSC: Lung squamous cell carcinoma LUAD: Lung adenocarcinoma LCC: Large cell carcinoma ADSQC: Adenosquamous carcinoma SCLC: Small cell carcinoma LCNEC: Large cell neuroendocrine carcinoma Sal. gl.-tp. carc.: Salivary gland type carcinoma Undiff. carc. Undifferentiated carcinoma

### Results of sequencing and associations with later metastasis

Among the 127 primary tumours with available sequencing results, 43.3% developed metastatic disease during follow-up. For *KRAS* and *EGFR*, the NGS and PCR results were combined and reported separately. For LUSC and LUAD, the clinicopathological characteristics of the sequenced cases were comparable to those of the nonsequenced cases (Online resource A9). The majority of patients had LUAD (54.3%) or LUSC (34.6%). The most common mutations in LUAD were in *TP53* (49.3%), *KRAS* (33.3%), *EGFR* (15.9%), *RB1* (8.7%), SETD2 (5.8%), *PTCH1*,* STK11* (each 4.3%) and *ATRX*,* BRCA2*,* CREBBP*,* MAP2K1*,* NOTCH3*, and *PIK3R1* (each 2.9%). In addition, we detected *ROS1* fusion in 4.3% of LUAD patients and *ALK*,* RET* and *MET* exon skipping in 1.4% of LUAD patients. The most common mutations in LUSC were in *TP53* (54.5%), *NFE2L2* (11.4%), *FBXW7*,* PTEN*,* RAD50*, and *PIK3CA* (6.8% each), and *ARID1A* and *SETD2* (4.6% each) **(**Table 2 and Online resource A10a and b).

For the remaining histological subtypes and for different stages, the number of sequenced cases was too low to analyse associations with metastasis.

In LUAD, metastasis was significantly associated with *CREBBP* mutations (HR 8.60, 95% CI 1.82–40.76, *p* = 0.007) and *MAP2K1* mutations (HR 5.88, 95% CI 1.30-26.63, *p* = 0.021). However, these mutations were rare, and the associations have wide confidence intervals, reflecting substantial statistical uncertainty. Mutations in *TP53* (HR 2.06, 95% CI 0.97–4.37, *p* = 0.059) and *STK11* (HR 3.57, 95% CI 0.82–15.59, *p* = 0.090) showed borderline statistical significance. Mutations in *MAP2K1* (HR 9.59, 95% CI 1.98–46.51, *p* = 0.005) were significantly associated with BM, whereas *CREBBP* showed a borderline association (HR 7.56, 95% CI 0.88–65.38, *p* = 0.066) **(**Table 2, Online resource A10a, b and A11). However, both findings were based on very small numbers of mutation-positive cases and should therefore be interpreted cautiously. *TP53* mutations appeared to be more common in LUAD patients who developed BM, although the difference was not statistically significant (see Kaplan-Meier plot Online resource Figure A2e).

In LUAD, *STK11* mutation-positive patients were more likely to develop ECM (HR 14.54, 95% CI 2.63–80.27, *p* = 0.002) (Online resource A11).

In LUSC, mutations in *NFE2L2* (HR 5.07, 95% CI 1.63–15.80, *p* = 0.005) were significantly associated with metastasis. In LUSC, *NFE2L2* also showed a trend toward an association with BM (HR 6.65, 95% CI 0.66–67.58, *p* = 0.109), whereas *FBXW7* mutations showed a borderline association with BM (HR 7.37, 95% CI 0.75–72.74, *p* = 0.087) (Table 2 and Online resource A10a and b). The latter findings were based on only a single mutation-positive BM case and should therefore be regarded as exploratory. However, mutation in *NFE2L2* was also associated with ECM (HR 4.69, 95% CI 1.27–17.26, *p* = 0.020), and this was based on three cases. *TP53* mutations appeared to be more common in LUSC patients who developed BM, although the difference was not statistically significant (see Kaplan‒Meier plot Online resource Figure A2f).

For results of NGS-testing stratified by P-stage and metastatic pattern in LUSC and LUAD, please see Online resource A10b.

No significant associations were found between the NGS results and early metastasis in LUAD or LUSC. However, *NFE2L2* mutations in LUSC (two of four, OR 12.00, 95% CI 0.71–203.14, *p* = 0.085) and *EGFR* L858R mutations in LUAD (two of nine, OR 8.00, 95% CI 0.68–94.66, *p* = 0.099) both showed borderline associations with early metastasis. Both analyses were based on very small numbers of cases and should be interpreted with considerable caution.

**Table 2:** NGS of the primary tumour, stratified by later metastasis status

Histopathology	Total tested	Brain metastasis	Total tested	Extracranial metastasis	Total tested	No metastasis
		Mutation		Mutation		Mutation
All	24		31		72	
LUAD	16/85	Mutation: TP53 (*n* = 10, 62.5%) KRAS (*n* = 6, 37.5%) EGFR (*n* = 3, 18.8%) MAP2K1, BRCA2 (*n* = 2, 12.5%) ATRX, BRCA1, CBL, CREBBP, NF1, PALB2, PTCH1, RB1, U2AF1 (*n* = 1, 6.3%)	13/127	Mutation: TP53 (*n* = 8, 61.5%) EGFR, KRAS, STK11 (*n* = 2, 15.4%) CREBBP, ERBB2, NOTCH3, RAD50, RB1, STAT3 (*n* = 1, 7.7%)	40/321	Mutation: TP53 (*n* = 16, 40%) KRAS (*n* = 15, 37.5%) EGFR (*n* = 6, 15.0%) RB1, SETD2 (*n* = 4, 10%) PIK3R1, PTCH1 (*n* = 2, 5%) ARID1A, ATRX, CDK4, ERBB2, IDH1, NOTCH1, NOTCH3, NRAS, STK11, TSC2 (*n* = 1, 2.5%) Negative (*n* = 5, 12.5%)
		CNV: TERT, RICTOR, EGFR (*n* = 1, 6.3%) Negative (*n* = 15, 93.8%)		CNV: AKT2, CCND1, MYC, FGF19, FGF3 (*n* = 1, 7.7%) Negative (*n* = 10, 76.9%)		CNV: AKT3, CDK6, FGFR1, PDGFRA, PIK3CA, KIT (*n* = 1, 2.5%) Negative (*n* = 36, 90.0%)
		Fusion: Negative (*n* = 16, 100%)		Fusion: ROS1 (*n* = 1, 7.7%) Negative (*n* = 12, 92.3%)		Fusion: ALK, MET exon 14 skipping, RET (*n* = 1, 2.5%) ROS1 (*n* = 2, 5.0%) Failed (*n* = 2, 5.0%) Negative (*n* = 33, 82.5%)
LUSC	4/13	Mutation: TP53 (*n* = 3, 75.0%) FBXW7, NFE2L2 (*n* = 1, 25.0%) Negative (*n* = 1, 25.0%)	13/75	Mutation: TP53 (*n* = 8, 61.5%) NFE2L2(*n* = 3, 23.1%) PIK3CA (*n* = 2, 15.4%) ARID1A, ATRX, CDKN2A, ESR1, FBXW7, RAD50, SETD2, SLX4, STK11, TSC1 (*n* = 1, 7.7%) Negative (*n* = 2, 15.4%)	27/212	Mutation: TP53 (*n* = 13, 48.2%) PTEN (*n* = 3, 11.1%) RAD50 (*n* = 2, 7.4%) ARID1A, FANCD2, FBXW7, KRAS, MET, NOTCH1, NFE2L2, PIK3CA, PTCH1, SETD2 (*n* = 1, 3.7%) Negative (*n* = 9, 33.3%)
		CNV: Negative (*n* = 4, 100%)		CNV: FGFR1 (*n* = 1, 7.7%) Failed (*n* = 1, 7.7%) Negative (*n* = 11, 84.6%)		CNV: Failed (*n* = 1, 3.7%) Negative (*n* = 26, 96.3%)
		Fusion: Negative (*n* = 4, 100%)		Fusion: Negative (*n* = 13, 100%)		Fusion: FGFR3 (*n* = 1, 3.7%) Failed (*n* = 1, 3.7%) Negative (*n* = 25, 92.6%)
ADSQC	0/4	–	1/7	Mutation and CNV: Failed (*n* = 1, 100%)	0/5	–
				Fusion: Negative (*n* = 1, 100%)		
LCC	0/3	–	2/11	Failed (*n* = 1, 50%) Negative (*n* = 1, 50%)	1/12	Mutation: PTEN (*n* = 1, 100%) TP53 (*n* = 1, 100%)
				CNV: Failed (*n* = 1, 50.0%) Negative (*n* = 1, 50.0%)		CNV: Negative (*n* = 1, 100%)
				Fusion: Negative (*n* = 1, 50%) Failed (*n* = 1, 50%)		Fusion: Negative (*n* = 1, 100%)
SCLC	0/4	–	0/2	–	0/7	–
LCNEC	3/3	Mutation: TP53 (*n* = 2, 66.7%) ATRX, MAP2K1, NF1, PALB2, SMARCA4 (*n* = 1, 33.3%) Negative (*n* = 1, 33.3%)	1/4	Mutation: STK11 (*n* = 1, 100%)	1/3	Mutation: ARID1A (*n* = 1, 100%) TP53 (*n* = 1, 100%)
		CNV: Negative (*n* = 3, 100%)		CNV: Negative (*n* = 1, 100%)		CNV: MYCL (*n* = 1, 100%) PDGFRA (*n* = 1, 100%)
		Fusion: Negative (*n* = 3, 100%)		Fusion: Negative (*n* = 1, 100%)		Fusion: Negative (*n* = 1, 100%)
Salivary gland-type carcinoma	0/0	–	1/2	Mutation: ERBB2 (*n* = 1, 100%) TP53 (*n* = 1, 100%)	3/3	Mutation: PTCH1 (*n* = 1, 33.3%) Negative (*n* = 2, 66.7%)
				CNV: CCNE1 (*n* = 1, 100%) AKT2 (*n* = 1, 100%)		CNV: Negative (*n* = 3, 100%)
				Fusion: Negative (*n* = 1, 100%)		Fusion: MYB (*n* = 1, 33.3%) Negative (*n* = 2, 66.7%)
Undifferentiated carcinoma	0/0	–	0/3	–	0/1	–
Mixed	1/1	Mutation, CNV and fusion: Negative (*n* = 1, 100%)	0/0	–	0/1	–
Other	0/0	–	0/0	–	0/3	–

Patients who did not develop metastasis, patients who developed brain metastasis and patients who developed extracranial metastasis. LUAD: Lung adenocarcinoma LUSC: Lung squamous cell carcinoma ADSQC: Adenosquamous carcinoma LCC: Large cell carcinoma SCLC: Small cell carcinoma LCNEC: Large cell neuroendocrine carcinoma

### Analysis of *EGFR* and *KRAS* mutations in adenocarcinoma and their associations with subsequent metastasis

By combining sequencing and PCR data, *EGFR* and *KRAS* mutation status was determined for 504 and 293 LUAD cases, respectively. Although *EGFR* and *KRAS* status were available for substantially larger numbers of patients than most other molecular alterations, several subtype-specific analyses remained based on small numbers of events and should therefore be interpreted with caution.

*EGFR* mutations were found in 11.9% of the patients. *EGFR* mutation was associated with female sex and strongly associated with never-smoking, and 54.7% of never-smoking women with LUAD had *EGFR* mutations. Among the patients with *EGFR* mutations, 43.3% developed metastatic disease, 38.5% of whom developed BM. Overall, *EGFR* positivity was not associated with overall metastasis or BM, but the subtype *EGFR* mutation L861Q was significantly associated with BM (HR 7.02, 95% CI 1.71–28.72, *p* = 0.007). However, this finding was based on only three patients, two of whom developed BM, and should therefore be interpreted with considerable caution (Online resource A12a, b and A13). *EGFR* positivity was associated with the development of BM in patients < 50 years of age and showed a borderline association in patients younger than 60 years of age (Table 3). However, these observations are based on small numbers and the younger patients had longer follow-up time. *EGFR* status was not associated with early metastasis or early BM (< 6 months). For results of *EGFR*-testing stratified by P-stage and metastatic pattern, please see Online resource A12b.

Following BM, *EGFR-*positive patients had significantly longer lung cancer-specific survival (HR 0.39, 95% CI 0.17–0.91, *p* = 0.029) and overall survial (HR 0.43, 95% CI 0.20–0.94, *p* = 0.035) than did *EGFR*-negative patients.

Although *EGFR* positivity was not associated with lung cancer-specific mortality, it was associated with a reduced risk of all-cause mortality (HR 0.65, 95% CI 0.44–0.98, *p* = 0.039). This may be attributable to a lower prevalence of smoking history among *EGFR-*positive patients (Online resource A12a and A13). However, the proportional hazards assumption was not fulfilled, indicating that the effect may vary over time and should therefore be interpreted with caution. Several significant associations observed with *EGFR* mutations became nonsignificant after adjustment for smoking status. This is likely attributable to the strong association between *EGFR* positivity and never-smoking status, suggesting potential multicollinearity in the model.

*KRAS* mutations occurred in 41.0% of the LUAD patients and were associated with smoking history, but not with age or sex. The *KRAS* G12C subtype was strongly associated with smoking (OR 14.46, 95% CI 1.93–108.06, *p* = 0.009). Overall, *KRAS* mutations were also associated with BM (HR 1.96, 95% CI 1.12–3.45, *p* = 0.019). Several *KRAS* subtypes also showed associations with metastatic outcomes, however, subtype-specific analyses were based on small numbers of patients and should therefore be interpreted cautiously (Online resource A14a, b and A15). *KRAS* status was not associated with early metastasis or early BM (< 6 months). For results of *KRAS*-testing stratified by P-stage and metastatic pattern, please see Online resource A14b.

Compared with wild-type mutations, *KRAS* mutations were associated with a significantly greater hazard for lung cancer-specific death (HR 1.44, 95% CI 1.00–2.07, *p* = 0.050). Interpretation of subtype-specific analyses was limited by the small number of cases in several mutation groups. Although some subtype analyses yielded statistically significant associations, these estimates were often accompanied by wide confidence intervals and should therefore be interpreted as exploratory observations rather than evidence of definitive biological differences between KRAS subtypes (Online resource A14a and A15). Several of these associations with mortality were attenuated after adjustment for smoking.

**Table 3:** Summary of associations with brain metastasis

Dependent variable	HR	95% CI	*p*-value
*Lower risk BM whole cohort*			
Age > 60	0.63	0.42–0.95	0.027
Age > 70	0.67	0.44–1.01	0.059
LUSC (compared to all)	0.26	0.15-0.047	< 0.001
*Higher risk BM whole cohort*			
Age < 60	1.59	1.05–2.41	0.027
Age < 70	1.49	0.99–2.25	0.059
*P-stage (compared to Ia)*			
Ib	1.72	0.92–3.24	0.092
IIa	2.63	1.40–4.95	0.003
IIb	3.06	1.49–6.27	0.002
IIIa	5.68	3.13–10.30	< 0.001
IIIb	14.39	1.90-108.73	0.010
*Histology (compared to LUSC)*			
Mixed	61.84	7.94-481.57	< 0.001
LCNEC	8.85	2.52–31.13	0.001
SCLC	6.88	2.24–21.09	0.001
Adsqc	6.71	2.19–20.58	0.001
LUAD	3.62	2.02–6.50	< 0.001
LCC	3.46	0.98–12.13	0.053
*Higher risk BM LUAD*			
Age < 70	1.58	0.98–2.54	0.063
Age < 50, +*EGFR* mutation*	7.08	1.81–27.78	0.005
Age < 60, +*EGFR* mutation*	2.95	0.83–10.54	0.095
*P-stage (compared to Ia)*			
Ib	2.00	1.00–4.00	0.049
IIa	2.39	1.12–5.09	0.024
IIb	4.20	1.94–9.06	0.001
IIIa	5.57	2.82–11.02	0.001
IIIb	121.16	14.28-1028.29	0.001
*KRAS*-mutation	1.96	1.12–3.45	0.019
*TP53*-mutation**			

*Stratified by EGFR-mutation. Associations with *p*-values between 0.05 and 0.10 were considered exploratory (borderline). ** Not statistically significant, but The Kaplan-Meier curves suggest a trend toward earlier BM in the TP53-mutant group, as indicated by the steeper early decline

### Paired sequencing of primary tumour and metastasis samples

We sequenced samples from 28 patients with paired primary and metastatic tumours, 23 of which had approved results for both tumour samples. Among the 28 patients, 16 had LUADs, six had LUSCs, two had LCCs, three had LCNECs, and one had mixed carcinoma. In LUAD patients, 12 of 16 patients had metastatic samples from the brain, and four of 16 had ECM. In LUSC, three of the six patients had metastatic samples from the brain, and the remaining three were ECM. In the paired sequencing analysis, *NFE2L2*,* FBXW7*,* KRAS*,* ERBB2*,* MAP2K1*,* CBL*, and *TP53* mutations were detected in both the primary and metastatic tumour in most cases. There were two patients with LCC and LUAD with *NF1* mutations in only the BM. In addition, one LUAD patient and one LCNEC patient presented with an *RB1* mutation only in the metastatic tumour; one of these metastatic tumours was a BM (LUAD) (Table 4).

**Table 4:** Paired sequencing of primary and metastatic tumour

Case	Histopathology	NGS primary tumour resection		Metastatic site	NGS metastasis	
		Mutation	CNV/Fusion		Mutation	CNV/Fusion
1	LUAD	*TP53*,* CBL*	Neg/Neg.	Brain	*TP53*,* NOTCH1*,* CBL*	Neg/Neg.
2	LCC	*TP53*,* NBN*	*FGFR1/Neg*	Brain	*TP53*,* NOTCH1*,* NF1*	Neg/Neg.
3	LUAD	Not done	N.d./N.d.	Other	*BRAF*,* PTEN*,* FBXW7*	Fail/Neg.
4	LUSC	*NFE2L2*,* TP53*,* FBXW7*	Neg/Neg.	Other	*NFE2L2*,* TP53*,* FBXW7*	Neg/Neg.
5	LUAD	Not done	N.d./N.d.	Brain	Fail	Fail/Fail
6	LUAD	*TP53*,* STK11*	Neg/Neg.	Other	*TP53*	Neg/Neg.
7	LUSC	*TP53*,* CDKN2A*,* ATRX*	Neg/Neg.	Other	*TP53*,* RAD51*	Neg/Neg.
8	LUAD	*BRCA1*	Neg/Neg.	Brain	*BRCA1*	Neg/Neg.
9	LUAD	*KRAS*	Neg/Neg.	Other	*KRAS*	Neg/Fail
10	LCNEC	*TP53*,* ATRX*,* MAP2K1*,* PALB2*	Neg/Neg.	Brain	Negative	Neg/Neg.
11	LUSC	Negative	Neg/Neg.	Brain	*TP53*,* FBXW7*,* NOTCH1*	Neg/Neg.
12	LUAD	*KRAS*	MDM2/Neg.	Brain	*KRAS*,* RB1*	Neg/Neg.
13	LUSC	Negative	Neg/Neg.	Other	Fail	Fail/Fail
14	LUAD	*KRAS*,* TP53*,* RB1*	Neg/Neg.	Brain	*KRAS*	Neg/Neg.
15	LCC	Negative	Neg/Neg.	Other	Negative	Neg/Neg.
16	LUAD	*KRAS*,* TP53*,* U2AF1*	Neg/Neg.	Brain	*KRAS*,* TP53*,* U2AF1*	Neg/Neg.
17	LUAD	*TP53*	Neg/Neg.	Brain	Negative	Neg/Neg.
18	LUAD	*BRCA2*,* KRAS*	Neg/Neg.	Brain	*KRAS*,* BRCA2*	Neg/Neg.
19	Mixed	Negative	Neg/Neg.	Brain	Not done	Not done
20	LUAD	*EGFR*,* TP53*,* PALB2*	Neg/Neg.	Brain	Fail	Neg/Fail
21	LUAD	*MAP2K1*,* TP53*,* ATRX*	Neg/Neg.	Brain	*MAP2K1*,* TP53*,* NF1*	Fail/Neg
22	LUAD	*ERBB2*	Neg/Neg.	Other	*ERBB2*,* TP53*	TERT/Neg
23	LUAD	*MAP2K1*,* TP53*,* ATRX*	Neg/Neg.	Brain	*MAP2K1*	Neg/Neg.
24	LUSC*	*TP53*	Neg/Neg.	Brain	Fail	Fail/Fail
25	LCNEC	*STK11*	Neg/Neg.	Other	*ARID1A*,* RB1*	Neg/Neg.
26	LCNEC	Negative	Neg/Neg.	Brain	*TP53*	Neg/Neg.
27	LUAD	*TP53*	Neg/Neg.	Brain	*TP53*	Neg/Neg.
28	LUSC	*NFE2L2*,* TP53*,* FBXW7*	Neg/Neg.	Brain	*TP53*,* NFE2L2*	Neg/Neg.

Comparison of the results of NGS for primary tumours and metastatic tumour from the same patient. *Patient 24: The primary tumour is LUSC, but the metastasis is ADSQC. LUAD: Lung adenocarcinoma LUSC: Lung squamous cell carcinoma ADSQC: Adenosquamous carcinoma LCC: Large cell carcinoma LCNEC: Large cell neuroendocrine carcinoma. Neg: Negative. N.d.: Not done

## Discussion

This study aimed to investigate the clinical, histopathological, and molecular factors associated with metastasis risk in patients with resected lung cancer, with a particular emphasis on BM. In this cohort of 912 patients with resected, stage I–III lung cancer, more than one-third (37.7%) developed metastatic disease during follow-up, and BM accounted for roughly one-third of these events. These findings reinforce that recurrence after curative-intent surgery remains common. While we confirmed associations with well-established risk factors such as stage, our results also suggest potential for further risk stratification on the basis of histopathological subtypes and specific molecular alterations. The relatively short median time to metastasis (1.48 years) and to BM specifically (1.01 years) underscores that most recurrences after resection occur early, within the first two postoperative years. A window that should inform the intensity and modality of surveillance imaging.

### Clinical and histopathological risk factors for metastasis

Both stage and histological subtype were strongly associated with metastatic risk. BM occurred more frequently in patients with LUAD, LCNEC, SCLC and ADSQC, as well as in those with more advanced disease (higher pT, pN, and overall P-stage). In contrast, BM was less common in LUSC. Compared with LUSC, LUAD was associated with a 3.62-fold greater hazard of developing BM. This observation is consistent with prior studies [22–25]. One study encompassing all disease stages reported that 43% of LUAD patients developed BM, whereas only 13% of LUSC patients did [22]. A meta-analysis by Wan et al. also revealed that LUSC was associated with a reduced risk of BM [26]. Furthermore, we observed that the hazard of BM increased with increasing P-stage in patients with LUAD, whereas no such stage-dependent trend was evident in patients with LUSC. However, the substantially smaller number of BM cases in LUSC (*n* = 13) than in LUAD (*n* = 85) may have limited the statistical power to detect such an association in the LUSC subgroup. While we were unable to demonstrate a stage-dependent increase in BM risk in LUSC, the observed association between advanced tumour stage and BM risk in LUAD is consistent with previous findings, which similarly reported the strongest association between P-stage and BM risk in LUAD [26].

In our analysis, we observed no significant associations between sex or smoking history and the occurrence of later BM. However, BM appeared to be more common in younger patients. Various studies have indicated a greater incidence of BM in younger patients [25, 27]. Younger patients are also more likely to harbour targetable alterations such as *ALK* fusion or *EGFR* mutations [27]. Johung et al. suggested that the higher prevalence of BM in patients with ALK fusions may reflect prolonged survival associated with effective tyrosine kinase inhibitor therapy, rather than an increased brain-metastatic propensity conferred by the fusion itself [28]. As our cohort consisted of patients with stage I–III disease and BM versus ECM was defined by the first site of recurrence following surgery, no patients received tyrosine kinase inhibitors before developing BM. Therefore, treatment-related prolongation of survival is unlikely to explain the increased frequency of BM among younger patients in this study. Furthermore, the BM cohort was not particularly enriched for *EGFR* mutations or *ALK* fusions. The younger patients had longer follow-up time, and therefore also more opportunities for BM to be detected. However, the median time to BM was approximately two months shorter in patients aged < 60 years than in those aged ≥ 60 years. If the observed association were solely explained by the longer follow-up time in younger patients, one might have expected a longer, rather than shorter, time to BM detection in this group. In addition, detection bias must also be considered, as younger patients may in practice receive closer clinical follow-up even though it is not in the guidelines. We cannot differentiate whether the observed association reflects an age-related biological difference in BM propensity, differences in follow-up duration, differences in clinical practice or a combination of these factors. Nevertheless, previous literature suggests that biological mechanisms may also contribute to the association between younger age and BM, including age-related differences in protein expression [26].

Another noteworthy observation was that the time from BM to lung cancer-specific or all-cause death was not associated with a higher original P-stage in patients with LUAD, as some higher P-stages were even associated with lower hazard ratios. These findings suggest that the original P-stage may have limited prognostic value once BM has developed. Similarly, previous studies have indicated that other factors, such as molecular characteristics, the ECM, and patient frailty, may play a more important role in determining outcomes at this stage of disease [29]. Although higher T- and N-stages have been associated with poorer prognosis after BM in other studies, those cohorts often included patients with stage IV disease, which may limit direct comparability of their findings to our population [25].

### Molecular risk factors for metastasis

We identified several molecular alterations that were associated with metastatic outcomes. While several investigated genes have an established biological relevance in NSCLC, several additional molecular associations were explored across multiple outcomes even with few observations. Therefore, findings involving infrequently altered genes should be interpreted cautiously and regarded as hypothesis-generating until they are validated in independent cohorts. Although insufficient to support firm conclusions on their own, these findings may provide valuable information when interpreted alongside data from other cohorts and future meta-analyses. Accordingly, the following discussion includes both statistically significant associations (*p* ≤ 0.05) and some predefined exploratory associations (*p* ≤ 0.10). Notably, many of these alterations may be associated with a limited number of biological pathways, particularly RAS/MAPK, PI3K/AKT/mTOR and NRF2-related signalling pathways. However, the present study was observational and did not directly assess pathway activation, transcriptional changes, protein expression, or functional effects of the identified alterations. Consequently, mechanistic interpretations should be considered exploratory and hypothesis-generating.

In LUAD, mutations in the *TP53* gene were borderline associated with overall metastasis, but not with BM specifically. Although the results of the formal test did not reach statistical significance, the Kaplan-Meier curves suggest a trend toward earlier BM in the *TP53*-mutant group, as indicated by the steeper early decline. *TP53* is a tumour suppressor gene that encodes the transcription factor protein p53. Complete functional loss is generally associated with mutation or inactivation affecting both alleles [6]. We were unable to determine whether a detected *TP53* mutation results in p53 inactivation, as we are unable to discern between homozygous and heterozygous mutations or identify alternative inactivating genetic mechanisms. The p53 protein plays a multifaceted role, influencing various biological pathways, with primary functions related to genome stability, apoptosis, and the regulation of the cell cycle and senescence. Mutations in *TP53* may therefore diminish tumour suppressor activity, but mutant p53 can also possess oncogenic properties itself [30]. In a meta-analysis, Gu et al. [31] reported that patients with stage I/II NSCLC and wild-type *TP53* had a significantly better prognosis with greater overall survival, particularly among those with LUAD. Saleh et al. [32] reported that truncating *TP53* mutations were negative prognostic factors in early-stage disease (I–IIIa), whereas no prognostic effect was observed in late-stage disease. Thus, although more knowledge is needed, *TP53* mutation might be a relevant prognostic factor. However, our study did not assess p53 function, allelic status, or downstream pathway activation, and therefore no mechanistic conclusions can be drawn from the observed associations. Saleh et al. also reported that *KEAP1* mutations were negative prognostic factors across both early- and late-stage disease. As *KEAP1* was not included in the Oncomine Comprehensive Assay v3 used in our study, we were unable to assess this association in our cohort.

In LUAD, *KRAS* mutations overall were associated with BM. Several individual *KRAS* subtypes also showed associations with BM, however, these analyses were based on small numbers of mutation-positive cases and often yielded wide confidence intervals. Therefore, the subtype-specific findings should be interpreted as exploratory observations rather than definitive associations. *KRAS* mutations are very common in LUAD, occurring in approximately 40% of LUAD cases, and represent major driver mutations that may affect several signalling pathways, including the RAS/MAPK/ERK and PI3K/AKT pathways. However, the prognostic significance of *KRAS* mutations in lung cancer is uncertain and may also differ between specific mutation subtypes [4, 33–37]. Isaksson et al. reported that G12C is associated with BM in a Swedish population-based study; however, patients with BM in that study had stage IV disease at diagnosis [38].

Although previous studies have associated *EGFR* mutations with advanced-stage disease and BM, we were unable to replicate the associations between *EGFR* mutations and BM in our cohort [39]. Comparisons with earlier research should nevertheless be made with caution, as definitions of BM may vary across studies, and in our cohort, BM was defined as the first metastatic lesion to occur.

Surprisingly, *EGFR-*positive patients did not have better lung cancer-specific survival, although they exhibited improved overall survival. The observed difference in overall survival appeared to be explained by differences in smoking history and not by age. As adjuvant tyrosine kinase inhibitor (TKI) therapy for early-stage *EGFR*-mutated NSCLC was not introduced in Norway until 2022, none of the patients in our cohort received adjuvant TKI treatment, and none received neoadjuvant therapy. The ADAURA trial demonstrated a 51% reduction in the risk of death among patients with *EGFR*-mutated NSCLC treated with adjuvant osimertinib [40]. Consequently, cancer-specific survival in this patient group is expected to improve in the coming years. The lower hazard of lung cancer-specific death and all cause death after BM in EGFR-mutant cases may partly be related to TKI treatment following metastatic recurrence. However, this analysis was based on relatively small numbers and should be interpreted cautiously.

We will also briefly comment on the mutations in *MAP2K1* and *CREBBP* that are associated with metastasis in LUAD. However, these findings were based on very small numbers of mutation-positive cases and were accompanied by wide confidence intervals. Although both genes have recognised biological roles in cancer development, the results should be regarded as hypothesis-generating [41–44].

In LUAD, we observed an association between *STK11* mutation and an increased risk of ECM. *STK11* is a tumour suppressor gene encoding the serine‒threonine kinase LKB1. Loss of functional LKB1 may result in aberrant activation of mTOR signalling and subsequent interaction with proteins in the PI3K pathway [45, 46]. *STK11* mutations have been proposed as a predictive biomarker of immunotherapy resistance in *KRAS*-mutated tumours, although some have suggested that they may instead represent a more broadly negative prognostic biomarker [4, 47, 48].

In our study, 11.4% of the tested LUSC patients harboured *NFE2L2* mutations. *NFE2L2* mutation was significantly associated with metastasis, showed a borderline association with early metastasis (< 6 months), and demonstrated a trend toward an association with BM (only one case). The *NFE2L2* gene encodes the transcription factor NRF2, a key regulator of the cellular antioxidant response. In normal cells, NRF2 protects against carcinogenesis induced by chemical or radiation-related damage. However, in cancer, persistent activation of the NRF2 pathway may promote several hallmarks of cancer, including tumour progression and metastasis [49–52]. We have not assessed whether NRF2 pathway activation occurred in the tumours included in this cohort. *KEAP1*, discussed above, functions as a negative regulator of NRF2 within the NRF2 antioxidant response pathway, and *KEAP1* mutations are more frequently observed in LUAD [52]. Although *NFE2L2* and *KEAP1* mutations are associated with increased programmed death-ligand 1 (PD-L1) expression and higher tumour mutational burden (TMB), patients harbouring these mutations often have poor clinical outcomes, even when treated with immunotherapy, suggesting that they may attenuate the predictive value of PD-L1 and TMB as biomarkers of immunotherapy response [53, 54].

In LUSC, *FBXW7* mutations showed a borderline association with BM. This observation was based on only a single mutation-positive case and should therefore be considered highly exploratory. Given the relatively low incidence of BM in LUSC, we will only briefly discuss the potential biological relevance of this finding. In a review by Wen et al. [55], multiple aspects of tumour biology, the tumour microenvironment, and BM development were described, and aberrant PI3K/AKT pathway activity and PTEN loss were identified in LUSC with BM in both primary and metastatic lesions. *FBXW7* mutations have been reported to interact with components of the PI3K/AKT pathway [45, 46, 56]. However, our data do not allow us to determine whether PI3K/AKT pathway activation was present in the *FBXW7*-mutated case in our cohort.

### Paired sequencing

Paired sequencing revealed a high degree of mutational concordance between primary tumours and their corresponding metastases. In particular, mutations in key oncogenes and tumour suppressor genes, including *NFE2L2*,* FBXW7*,* KRAS*,* ERBB2*,* MAP2K1*,* CBL* and *TP53*, were consistently detected in both primary and metastatic lesions, with only a few notable exceptions. In our cohort, nucleic acids extracted from primary tumours were of high quality, whereas material from the corresponding metastases was generally more degraded. Despite the generally lower quality of metastatic samples, the high level of concordance observed in our cohort was consistent with previous reports, particularly for established driver mutations. However, apparent discrepancies should be interpreted with caution, as they may partly reflect differences in sample quality [57–60]. Lee et al. [61] performed comprehensive whole-exome sequencing of 35 paired primary NSCLC and metastatic tumours and similarly reported a high degree of concordance, with an average of 67% shared mutations between primary and metastatic lesions. On the basis of the extensive overlap in mutational profiles, they suggested that metastatic dissemination occurred relatively late during tumour evolution. Likewise, Andrews et al. [62] reported concordance for 63% of mutations identified in BM compared with matched primary NSCLC tumours, with *TP53* (67%), *KRAS* (66%) and *EGFR* (58%) representing the most frequent alterations.

In the paired sequencing analysis, we identified two cases (one LCC and one LUAD), in which an *NF1* mutation was detected exclusively in the BM. The *NF1* gene encodes neurofibromin, a negative regulator of RAS signalling. Consequently, loss‑of‑function mutations in *NF1* may lead to hyperactivation of the RAS/MAPK signalling pathway. Given the extensive crosstalk between the RAS/MAPK and PI3K pathways, *NF1* inactivation may also indirectly promote PI3K pathway activation [63]. However, this observation was based on only two cases and should therefore be considered hypothesis-generating. We have no functional data demonstrating pathway activation in these tumours, and no conclusions regarding the biological significance of *NF1* mutations in BM can therefore be drawn from our findings.

The potential involvement of PI3K signalling is supported by findings reported by Wang et al., who sequenced 61 paired primary NSCLC tumours and BM [60]. They observed a high degree of concordance between primary tumours and BMs; however, copy number variations were more frequent in the metastatic lesions. Furthermore, mutations affecting the *CDK4/CCND1* and *CDKN2A/2B* axes, as well as genes involved in PI3K signalling, were more common in metastatic lesions. Notably, patients harbouring PI3K pathway-activating alterations in the primary tumour developed BM significantly earlier than non‑carriers did.

### Strengths and limitations

The study has several strengths. The cohort included the majority of patients with surgically resected lung cancer in the largest health region in Norway, comprising 3.1 million inhabitants in 2025. The dataset is comprehensive and includes clinical, histopathological and sequencing data. The follow-up was conducted by a dedicated research nurse. We consider outcome misclassification bias to be small, owing to almost complete recurrence data over a median follow-up time exceeding five years and the use of unique personal identification numbers that ensure direct linkage to death registry data.

There are several limitations in our study. First, the sequencing data were only available for a nonrandom subset of the cohort. The limited size of the subset population may have reduced the ability to detect associations with certain mutations because of insufficient statistical power (type II error). We therefore discuss the relevance of nonsignificant findings, particularly when the observed effects were of a magnitude that could be clinically relevant. While analyses based on a nonrandomly selected subset of the cohort may limit the generalisability of the results, selection into the sequencing subset was driven by tissue availability and inclusion in previous molecular studies rather than metastatic outcome. Furthermore, the sequenced LUAD and LUSC cases were broadly comparable to the nonsequenced cases with respect to major clinicopathological characteristics. We therefore consider the risk of substantial selection bias in the estimated associations between mutations and later metastasis to be limited. Multiple comparisons were performed across several genes, outcomes, subtypes, and subgroup analyses without formal adjustment for multiplicity. Consequently, the risk of type I error is increased, and some statistically significant associations may represent chance findings rather than true biological effects. Therefore, these results should be interpreted with caution and considered primarily hypothesis-generating. Furthermore, we report findings with *p*-value > 0.05 and ≤ 0.1 to highlight potential trends. However, these observations should be interpreted with even greater caution and not be regarded as statistically significant evidence [64–66].

Second, the study population included only surgically treated lung cancer patients, and the results might therefore not be generalisable to nonresectable cases. The Norwegian guidelines for lung cancer suggest surgical resection for patients with cT1-3, N0-1, M0, and in special cases with T4-disease. The most important reasons for not undergoing surgery in patients with early-stage disease are advanced age and comorbidities. We consider it likely that frequencies, survival outcomes, and associations of interest may differ in patients who do not undergo surgical resection owing to older age, a greater burden of comorbidity, and the absence of curative surgical treatment. Another consideration is that Norwegian guidelines were updated regularly during the study period. While we cannot exclude temporal effects in eligibility for surgery, our results should be considered the overall effect of the decision to perform surgery during the period 2006–2018.

Third, an important limitation is the heterogeneity of the study population. Although the study focuses on surgically resected lung cancer, the cohort included patients with pathological stages I–III. Stage III disease differs substantially from stage I disease with respect to biological behaviour, recurrence risk, prognosis and treatment recommendations. Consequently, some observed associations may partly reflect differences in stage distribution rather than intrinsic metastatic potential. The findings should therefore be interpreted as applying to surgically resected stage I–III lung cancer rather than to a homogeneous early-stage population.

Fourth, data for follow-up assessments were collected by a study research nurse primarily from records at Oslo University Hospital and supplemented by local hospitals. While this approach suggests a strong follow-up protocol, missing data cannot be entirely excluded. Patient transfer to nursing care facilities and other reasons for nonadherence to surveillance protocols may have introduced detection bias through delayed or missed detection of metastases, potentially resulting in outcome misclassification.

Fifth, for primary tumour we could extract high-quality DNA and RNA from fresh frozen tissue, but for metastatic tumours only FFPE blocks were available. FFPE samples exhibit more noise/errors from false mapping, and findings can also be rejected or missed if the predefined quality parameters are not met. Interpretation of the results of paired sequencing should therefore be performed with caution. However, the risk of false positive sequencing results is limited, and we consider identical mutations in both the primary and metastatic tumour as relevant findings.

Sixth, it is important to recognize that targeted sequencing provides limited insight into the full spectrum of molecular events occurring within tumour cells. Pathway activation can occur through various mechanisms, including methylation and epigenetic modifications, which are not detectable using this method [67–70].

Seventh, BRG1 immunohistochemistry was not routinely performed during the study period. Therefore, tumours that would currently be classified as *SMARCA4*-deficient thoracic tumours in the 5th edition of the WHO Classification of Thoracic Tumours, could not be specifically identified [71]. As a result, misclassification of a small number of undifferentiated carcinomas cannot be excluded.

Eighth, because brain MRI was generally performed in response to clinical suspicion of BM, asymptomatic BM may not have been detected until symptoms developed. This symptom-triggered imaging strategy may therefore have introduced detection, surveillance, and outcome misclassification bias. Patients with longer follow-up, such as younger patients in this cohort, may have had greater opportunities for BM detection. Consequently, the observed association between younger age and radiologically detected BM may reflect both biological differences and differences in surveillance and detection. In addition, the recorded date of BM diagnosis reflects the time of radiological detection rather than the true biological onset of intracranial metastatic disease. Small or asymptomatic metastases may therefore have been present for a variable period before becoming clinically apparent. Furthermore, imaging practices, MRI protocols, scanner performance, and thresholds for obtaining brain imaging may have varied across institutions and throughout the study period. Therefore, the reported associations should be interpreted as relating to radiologically detected BM rather than the biological initiation of metastatic spread.

Ninth, we do not have complete record of which patients received adjuvant therapy or systemic/local treatment when they progressed. As described under Methods (Patients), we know that no patients received neoadjuvant therapy, we know which patients that were offered adjuvant therapy according to guidelines and we know when targeted- or immunotherapy was introduced in late-stage disease. However, we have no complete records of which patients received treatment and have therefore not included treatment exposure as a covariate in analyses. It is likely that these factors are important confounders, since adjuvant treatment may have influenced the likelihood and timing of subsequent metastatic disease and survival, while treatments administered after metastatic occurrence may have influenced survival. Furthermore, the cohort was treated surgically between 2006 and 2018, before the widespread use of contemporary perioperative targeted therapy and immunotherapy. Therefore, it is necessary to validate our results is larger cohorts for more robustness, and with contemporary comprehensive treatment data included.

Tenth, ECOG performance status was not systematically recorded at baseline or at the time of BM. Although the cohort consisted of patients selected for curative-intent surgical treatment, and patients with poor performance status would generally not have been offered such treatment according to Norwegian lung cancer guidelines, individual performance-status measurements were unavailable [10]. Performance status may also have changed during follow-up. Its absence may therefore have resulted in confounding, particularly in analyses of survival after BM since performance status may influence both the likelihood of receiving treatment and survival after BM, independently of tumour stage and molecular characteristics.

Eleventh, the histopathological composition of our cohort may limit its representativeness and generalisability. LUAD accounted for 58.4% of cases and LUSC for 32.9%. In a historical national surgical cohort reported by the Norwegian Lung Cancer Registry in 2013–2014, LUAD accounted for 62.1% and LUSC for 26.9% of 531 surgically resected patients after exclusion of carcinoid tumours [72]. The proportion of LUSC was therefore somewhat higher in our cohort. The cohort was recruited from Oslo University Hospital in the South-Eastern region of Norway, including Oslo and surrounding urban areas. Historical Norwegian data demonstrate substantial regional variation in smoking prevalence, with high smoking prevalence in our cohorts region in the 1970s until the middle of 1990s [73]. Regional differences in smoking prevalence may have contributed to the observed relatively high proportion of LUSC in our cohort, since smoking exposure is so closely connected to LUSC.

Finally, event rates for metastasis and lung cancer death were insufficient for Kaplan-Meier median estimates to be reached within the follow-up period. We therefore reported median times to event among patients who experienced the event during follow-up. These descriptive measures complement the Kaplan-Meier and Cox regression analyses, which properly incorporate censoring, but should not be interpreted as estimates of prognosis for the cohort as a whole.

## Summary/conclusion

In this cohort of patients with resected, stage I–III lung cancer, the risk of BM was associated with younger age, increased pathological tumour and lymph-node stage and histopathological subtypes including LUAD, LCNEC, SCLC, and ADSQC patients, whereas LUSC was associated with a lower risk of BM. Pathological stage appeared to have limited prognostic value after the development of BM in patients with LUAD.

Several molecular alterations were associated with BM. The most consistent findings involved *KRAS* mutations in LUAD. Additional associations involving specific *KRAS* and *EGFR* subtypes and rarer molecular alterations were observed, but these analyses were based on small numbers of cases and should therefore be considered exploratory. In addition, ECM was associated with *STK11* mutation in LUAD and *NFE2L2* mutation in LUSC. Several of the identified alterations may be related to the RAS/MAPK, PI3K/AKT/mTOR, or NRF2 pathways, suggesting that these pathways warrant further investigation in metastatic progression. However, the present observational study cannot establish pathway activation or causality.

Paired sequencing demonstrated a high degree of mutational concordance between primary tumours and corresponding metastatic lesions, suggesting that major driver alterations may be retained during metastatic dissemination. These findings should nevertheless be interpreted cautiously because the number of paired samples was limited and metastatic specimens were generally derived from FFPE samples.

These findings require validation in larger, independent, stage-stratified and contemporary cohorts in which current treatment exposures are comprehensively recorded and incorporated into the analyses. Future studies using more extensive molecular profiling, including assessment of biallelic tumour-suppressor-gene inactivation, transcriptomic and epigenetic alterations, and the tumour microenvironment, may improve understanding of metastatic progression and support the development of more precise risk-stratification models for BM and ECM. Active and continuous surveillance with liquid biopsy and MRI of the brain, could also lead to more precise timing and less potential detection bias in a future cohort study. In particular, younger patients with LUAD or patients harbouring *KRAS* or *TP53* mutations, as well as patients with LUSC and *NFE2L2* mutations may represent relevant groups for evaluation in future validation studies with emphasis on risk of BM. These subgroups may benefit from frequent cranial MRI surveillance rather than solely CT scans of the thorax. Furthermore, patients with *LUAD* and *STK11*-mutation and LUSC patients with *NFE2L2*-mutation might benefit of closer or different follow up to detect ECM. However, all molecular findings reported here should be regarded as hypothesis-generating and should not be interpreted as a recommendation for intensified surveillance or altered treatment in current clinical practice.

## Supplementary Information

Below is the link to the electronic supplementary material.

Supplementary Material 1

## Data Availability

Some data generated or analysed during this study are included in this published article and supplementary files. Further datasets are not publicly available. However, they can be made available from the corresponding author on reasonable request and after approval by the Regional Ethics Committee.

## References

[1] Cancer Registry of Norway (2025) Cancer in Norway 2024: Cancer incidence, mortality, survival and prevalence in Norway. https://www.fhi.no/en/publ/2025/cancer-in-norway-2024/. Accessed 05 Oct 2026

[2] Ji Y, Zhang Y, Liu S, Li J, Jin Q, Wu J, Duan H, Liu X, Yang L, Huang Y (2025) The epidemiological landscape of lung cancer: current status, temporal trend and future projections based on the latest estimates from GLOBOCAN 2022. J Natl Cancer Cent 5:278–286. 10.1016/j.jncc.2025.01.003 PMC12276566 40693237

[3] Norwegian Institute of Public Health; Cancer Registry of Norway (2025) Årsrapport 2024 med resultater og forbedringstiltak fra Nasjonalt kvalitetsregister for lungekreft. https://www.fhi.no/contentassets/b19a8ea7ddb74efc92ad44cf16d74c5d/arsrapport-2024-nasjonalt-kvalitetsregister-for-lungekreft.pdf. Accessed 02 Oct 2026

[4] Garinet S, Wang P, Mansuet-Lupo A, Fournel L, Wislez M, Blons H (2022) Updated prognostic factors in localized NSCLC. Cancers 14:1400. 10.3390/cancers14061400 PMC8945995 35326552

[5] Sacks P, Rahman M (2020) Epidemiology of brain metastases. Neurosurg Clin N Am 31:481–488. 10.1016/j.nec.2020.06.001 32921345

[6] Weinberg RA (2006) The biology of cancer. WW Norton & Company, New York

[7] Howlader N, Forjaz G, Mooradian MJ, Meza R, Kong CY, Cronin KA, Mariotto AB, Lowy DR, Feuer EJ (2020) The effect of advances in lung-cancer treatment on population mortality. N Engl J Med 383:640–649. 10.1056/NEJMoa1916623 PMC8577315 32786189

[8] Lu T, Yang X, Huang Y, Zhao M, Li M, Ma K, Yin J, Zhan C, Wang Q (2019) Trends in the incidence, treatment, and survival of patients with lung cancer in the last four decades. Cancer Manag Res 11:943–953. 10.2147/cmar.S187317 PMC6345192 30718965

[9] Zer A, Ahn MJ, Barlesi F, Bubendorf L, De Ruysscher D, Garrido P, Gautschi O, Hendriks LE, Jänne PA, Kerr KM, Mascaux C et al (2025) Early and locally advanced non-small-cell lung cancer: ESMO Clinical Practice Guideline for diagnosis, treatment and follow-up. Ann Oncol 36:1245–1262. 10.1016/j.annonc.2025.08.003 40885528

[10] Helsedirektoratet (2025) Lungekreft, mesoteliom og thymom: handlingsprogram. https://www.helsedirektoratet.no/retningslinjer/lungekreft-mesoteliom-og-thymom-handlingsprogram/innledning#revisjon-mai-2025. Accessed 02 October 2026

[11] Huang RSP, Harries L, Decker B, Hiemenz MC, Murugesan K, Creeden J, Tolba K, Stabile LP, Ramkissoon SH, Burns TF, Ross JS (2022) Clinicopathologic and genomic landscape of non-small cell lung cancer brain metastases. Oncologist 27:839–848. 10.1093/oncolo/oyac094 PMC9526503 35598205

[12] Travis WD, Brambilla E, Burke AP, Marx A, Nicholson AG (2015) WHO classification of tumours of the lung, pleura, thymus and heart, 4th edition. International Agency for Research on Cancer (IARC), Lyon 10.1097/JTO.0000000000000663 26291007

[13] La Salvia A, Meyer ML, Hirsch FR, Kerr KM, Landi L, Tsao MS, Cappuzzo F (2024) Rediscovering immunohistochemistry in lung cancer. Crit Rev Oncol Hematol 200:104401. 10.1016/j.critrevonc.2024.104401 38815876

[14] Lund-Iversen M, Grøholt KK, Helland Å, Borgen E, Brustugun OT (2015) NUT expression in primary lung tumours. Diagn Pathol 10:156–156. 10.1186/s13000-015-0395-9 PMC4560868 26341600

[15] George J, Walter V, Peifer M, Alexandrov LB, Seidel D, Leenders F, Maas L, Müller C, Dahmen I, Delhomme TM, Ardin M et al (2018) Integrative genomic profiling of large-cell neuroendocrine carcinomas reveals distinct subtypes of high-grade neuroendocrine lung tumors. Nat Commun 9:1048. 10.1038/s41467-018-03099-x PMC5849599 29535388

[16] Norwegian Institute of Public Health Norwegian Cause of Death Registry (2026) https://www.fhi.no/en/ch/cause-of-death-registry/. Accessed 02 Oct 2026

[17] Dyrbekk APH, Warsame AA, Suhrke P, Ludahl MO, Moe JO, Eide IJZ, Lund-Iversen M, Brustugun OT (2023) Evaluation of ROS1 expression and rearrangements in a large cohort of early-stage lung cancer. Diagn Pathol 18:70. 10.1186/s13000-023-01357-1 PMC10224579 37237384

[18] Dyrbekk APH, Warsame AA, Suhrke P, Ludahl MO, Zecic N, Moe JO, Lund-Iversen M, Brustugun OT (2024) Evaluation of NTRK expression and fusions in a large cohort of early-stage lung cancer. Clin Exp Med 24:10. 10.1007/s10238-023-01273-PMC10798916 38240952

[19] Tamborero D, Dienstmann R, Rachid MH, Boekel J, Baird R, Braña I, De Petris L, Yachnin J, Massard C, Opdam FL, Schlenk R et al (2020) Support systems to guide clinical decision-making in precision oncology: the Cancer Core Europe Molecular Tumor Board Portal. Nat Med 26:992–994. 10.1038/s41591-020-0969-2 32632195

[20] Li MM, Datto M, Duncavage EJ, Kulkarni S, Lindeman NI, Roy S, Tsimberidou AM, Vnencak-Jones CL, Wolff DJ, Younes A, Nikiforova MN (2017) Standards and guidelines for the interpretation and reporting of sequence variants in cancer: a joint consensus recommendation of the Association for Molecular Pathology, American Society of Clinical Oncology, and College of American Pathologists. J Mol Diagn 19:4–23. 10.1016/j.jmoldx.2016.10.002 PMC5707196 27993330

[21] StataCorp LCC (2023) Stata statistical software: release 18

[22] Mujoomdar A, Austin JH, Malhotra R, Powell CA, Pearson GD, Shiau MC, Raftopoulos H (2007) Clinical predictors of metastatic disease to the brain from non-small cell lung carcinoma: primary tumor size, cell type, and lymph node metastases. Radiology 242:882–888. 10.1148/radiol.2423051707 17229875

[23] Xu X, Chen G, Fan S, Zhang Q, Huang W, Chen J, Ji W, Ma J, He J (2024) Risk factors for brain metastases in locally advanced non-small cell lung cancer patients treated with radical radiotherapy. J Thorac Dis 16:479–490. 10.21037/jtd-23-1435 PMC10894422 38410550

[24] Shi AA, Digumarthy SR, Temel JS, Halpern EF, Kuester LB, Aquino SL (2006) Does initial staging or tumor histology better identify asymptomatic brain metastases in patients with non-small cell lung cancer? J Thorac Oncol 1:205–210. 10.1016/s1556-0864(15)31569-0 17409858

[25] Hao Y, Li G (2023) Risk and prognostic factors of brain metastasis in lung cancer patients: a surveillance, epidemiology, and end results population-based cohort study. Eur J Cancer Prev 32:498–511. 10.1097/cej.0000000000000790 PMC10373853 36804864

[26] Wan X, Mo H, Chen J, Zhou Q, Qiu S, Zou Y, Wu W, Tang Q, Wang S (2025) Risk factors for brain metastasis in lung cancer: an umbrella review of systematic reviews and meta-analyses. BMJ Open 15:e087181. 10.1136/bmjopen-2024-087181 PMC11749772 39773793

[27] Suidan AM, Roisman L, Belilovski Rozenblum A, Ilouze M, Dudnik E, Zer A, Peled N (2019) Lung cancer in young patients: higher rate of driver mutations and brain involvement, but better survival. J Glob Oncol 5:1–8. 10.1200/jgo.18.00216 PMC6550091 31067141

[28] Johung KL, Yeh N, Desai NB, Williams TM, Lautenschlaeger T, Arvold ND, Ning MS, Attia A, Lovly CM, Goldberg S, Beal K et al (2016) Extended survival and prognostic factors for patients with ALK-rearranged non-small-cell lung cancer and brain metastasis. J Clin Oncol 34:123–129. 10.1200/jco.2015.62.0138 PMC5070549 26438117

[29] Sperduto PW, Mesko S, Li J, Cagney D, Aizer A, Lin NU, Nesbit E, Kruser TJ, Chan J, Braunstein S, Lee J et al (2020) Survival in patients with brain metastases: summary report on the updated diagnosis-specific graded prognostic assessment and definition of the eligibility quotient. J Clin Oncol 38:3773–3784. 10.1200/jco.20.01255 PMC7655019 32931399

[30] Wang H, Guo M, Wei H, Chen Y (2023) Targeting p53 pathways: mechanisms, structures, and advances in therapy. Signal Transduct Target Ther 8:92. 10.1038/s41392-023-01347-1 PMC9977964 36859359

[31] Gu J, Zhou Y, Huang L, Ou W, Wu J, Li S, Xu J, Feng J, Liu B (2016) TP53 mutation is associated with a poor clinical outcome for non-small cell lung cancer: evidence from a meta-analysis. Mol Clin Oncol 5:705–713. 10.3892/mco.2016.1057 PMC5228103 28101350

[32] Saleh MM, Scheffler M, Merkelbach-Bruse S, Scheel AH, Ulmer B, Wolf J, Buettner R (2022) Comprehensive analysis of TP53 and KEAP1 mutations and their impact on survival in localized- and advanced-stage NSCLC. J Thorac Oncol 17:76–88. 10.1016/j.jtho.2021.08.764 34601169

[33] Shepherd FA, Domerg C, Hainaut P, Jänne PA, Pignon JP, Graziano S, Douillard JY, Brambilla E, Le Chevalier T, Seymour L, Bourredjem A et al (2013) Pooled analysis of the prognostic and predictive effects of KRAS mutation status and KRAS mutation subtype in early-stage resected non-small-cell lung cancer in four trials of adjuvant chemotherapy. J Clin Oncol 31:2173–2181. 10.1200/jco.2012.48.1390 PMC4881333 23630215

[34] Izar B, Zhou H, Heist RS, Azzoli CG, Muzikansky A, Scribner EE, Bernardo LA, Dias-Santagata D, Iafrate AJ, Lanuti M (2014) The prognostic impact of KRAS, its codon and amino acid specific mutations, on survival in resected stage I lung adenocarcinoma. J Thorac Oncol 9:1363–1369. 10.1097/jto.0000000000000266 25122432

[35] Yu HA, Sima CS, Shen R, Kass S, Gainor J, Shaw A, Hames M, Iams W, Aston J, Lovly CM, Horn L et al (2015) Prognostic impact of KRAS mutation subtypes in 677 patients with metastatic lung adenocarcinomas. J Thorac Oncol 10:431–437. 10.1097/jto.0000000000000432 PMC4479120 25415430

[36] Adderley H, Blackhall FH, Lindsay CR (2019) KRAS-mutant non-small cell lung cancer: converging small molecules and immune checkpoint inhibition. EBioMedicine 41:711–716. 10.1016/j.ebiom.2019.02.049 PMC6444074 30852159

[37] Khadse A, Haakensen VD, Silwal-Pandit L, Hamfjord J, Micke P, Botling J, Brustugun OT, Lingjærde OC, Helland Å, Kure EH (2022) Prognostic significance of the loss of heterozygosity of KRAS in early-stage lung adenocarcinoma. Front Oncol 12:873532. 10.3389/fonc.2022.873532 PMC9098994 35574381

[38] Isaksson J, Berglund A, Louie K, Willén L, Hamidian A, Edsjö A, Enlund F, Planck M, Vikström A, Johansson M, Hallqvist A et al (2023) KRAS G12C mutant non-small cell lung cancer linked to female sex and high risk of CNS metastasis: population-based demographics and survival data from the National Swedish Lung Cancer Registry. Clin Lung Cancer 24:507–518. 10.1016/j.cllc.2023.05.002 37296038

[39] Zhang J, Luo Z, Xie Z, Huang J, Lin H, Pan H, Chen L, Wu C, Wu L, Zhou Y, Zheng J et al (2025) Association between EGFR mutation types and incidence of brain metastases in postoperative patients with stage I–III NSCLC. Tumori 111:200–209. 10.1177/03008916251343724 40411223

[40] Tsuboi M, Herbst RS, John T, Kato T, Majem M, Grohé C, Wang J, Goldman JW, Lu S, Su W-C, de Marinis F et al (2023) Overall survival with osimertinib in resected EGFR-mutated NSCLC. N Engl J Med 389:137–147. 10.1056/NEJMoa2304594 37272535

[41] Arcila ME, Drilon A, Sylvester BE, Lovly CM, Borsu L, Reva B, Kris MG, Solit DB, Ladanyi M (2015) MAP2K1 (MEK1) mutations define a distinct subset of lung adenocarcinoma associated with smoking. Clin Cancer Res 21:1935–1943. 10.1158/1078-0432.Ccr-14-2124 PMC4401580 25351745

[42] de Jager VD, Timens W, Bayle A, Botling J, Brcic L, Büttner R, Fernandes MGO, Havel L, Hochmair M, Hofman P, Janssens A et al (2024) Future perspective for the application of predictive biomarker testing in advanced stage non-small cell lung cancer. Lancet Reg Health Eur 38:100839. 10.1016/j.lanepe.2024.100839 PMC10928270 38476751

[43] Cheng ML, Lee JK, Kumar R, Klein H, Raskina K, Schrock AB, Michael KS, Mazor T, Cerami E, Oxnard GR, Liu D et al (2022) Response to MEK inhibitor therapy in MAP2K1 (MEK1) K57N non-small-cell lung cancer and genomic landscape of MAP2K1 mutations in non-small-cell lung cancer. JCO Precis Oncol 6:e2200382. 10.1200/po.22.00382 36455195

[44] Jia D, Augert A, Kim DW, Eastwood E, Wu N, Ibrahim AH, Kim KB, Dunn CT, Pillai SPS, Gazdar AF, Bolouri H et al (2018) Crebbp loss drives small cell lung cancer and increases sensitivity to HDAC inhibition. Cancer Discov 8:1422–1437. 10.1158/2159-8290.Cd-18-0385 PMC6294438 30181244

[45] Laderian B, Mundi P, Fojo T, Bates E (2020) Emerging therapeutic implications of STK11 mutation: case series. Oncologist 25(9):733–737. 10.1634/theoncologist.2019-0846 PMC7485370 32396674

[46] Zhou W, Marcus AI, Vertino PM (2013) Dysregulation of mTOR activity through LKB1 inactivation. Chin J Cancer 32:427–433. 10.5732/cjc.013.10086 PMC3845579 23668926

[47] Skoulidis F, Goldberg ME, Greenawalt DM, Hellmann MD, Awad MM, Gainor JF, Schrock AB, Hartmaier RJ, Trabucco SE, Gay L, Ali SM et al (2018) STK11/LKB1 mutations and PD-1 inhibitor resistance in KRAS-mutant lung adenocarcinoma. Cancer Discov 8:822–835. 10.1158/2159-8290.Cd-18-0099 PMC6030433 29773717

[48] Di Federico A, De Giglio A, Parisi C, Gelsomino F (2021) STK11/LKB1 and KEAP1 mutations in non-small cell lung cancer: prognostic rather than predictive? Eur J Cancer 157:108–113. 10.1016/j.ejca.2021.08.011 34500370

[49] Smolkova K, Miko E, Kovacs T, Leguina-Ruzzi A, Sipos A, Bai P (2020) Nuclear factor erythroid 2-related factor 2 in regulating cancer metabolism. Antioxid Redox Signal 33:966–997. 10.1089/ars.2020.8024 PMC7533893 31989830

[50] Rojo de la Vega M, Chapman E, Zhang DD (2018) NRF2 and the hallmarks of cancer. Cancer Cell 34:21–43. 10.1016/j.ccell.2018.03.022 PMC6039250 29731393

[51] Satpathy S, Krug K, Jean Beltran PM, Savage SR, Petralia F, Kumar-Sinha C, Dou Y, Reva B, Kane MH, Avanessian SC, Vasaikar SV et al (2021) A proteogenomic portrait of lung squamous cell carcinoma. Cell 184(4348–4371):e40. 10.1016/j.cell.2021.07.016 PMC8475722 34358469

[52] Sanchez-Ortega M, Carrera AC, Garrido A (2021) Role of NRF2 in lung cancer. Cells 10:1879. 10.3390/cells10081879 PMC8394503 34440648

[53] Duan J, Zhang Y, Chen R, Liang L, Huo Y, Lu S, Zhao J, Hu C, Sun Y, Yang K, Chen M et al (2023) Tumor-immune microenvironment and NRF2 associate with clinical efficacy of PD-1 blockade combined with chemotherapy in lung squamous cell carcinoma. Cell Rep Med 4:101302. 10.1016/j.xcrm.2023.101302 PMC10772345 38052215

[54] Singh A, Daemen A, Nickles D, Jeon SM, Foreman O, Sudini K, Gnad F, Lajoie S, Gour N, Mitzner W, Chatterjee S et al (2021) NRF2 activation promotes aggressive lung cancer and associates with poor clinical outcomes. Clin Cancer Res 27:877–888. 10.1158/1078-0432.CCR-20-1985 PMC10867786 33077574

[55] Wen J, Yu JZ, Liu C, Ould Ismail AAO, Ma W (2024) Exploring the molecular tumor microenvironment and translational biomarkers in brain metastases of non-small-cell lung cancer. Int J Mol Sci 25:2044. 10.3390/ijms25042044 PMC10889194 38396722

[56] Shen W, Zhou Q, Peng C, Li J, Yuan Q, Zhu H, Zhao M, Jiang X, Liu W, Ren C (2022) FBXW7 and the hallmarks of cancer: underlying mechanisms and prospective strategies. Front Oncol 12:880077. 10.3389/fonc.2022.880077 PMC9063462 35515121

[57] Xie T, Liu Z, Li Y, Wang S, Zhai Y, Teng F, Hao X, Wang Y, Wang H, Zhang X, Wu X et al (2023) Evolutionary characteristics and immunologic divergence of lung and brain metastasis lesions in NSCLC. Mol Cancer Res 21:374–385. 10.1158/1541-7786.Mcr-22-0474 PMC10068444 36534728

[58] Werner RS, Rechsteiner M, Moch H, Curioni-Fontecedro A, Weller M, Weiss T, Regli L, Le Rhun E, Mairinger F, Opitz I, Soltermann A (2024) Genetic profiles of oligometastatic non-small-cell lung cancer and corresponding brain metastases. Eur J Cardiothorac Surg. 10.1093/ejcts/ezae217 PMC11162753 38796684

[59] Shih DJH, Nayyar N, Bihun I, Dagogo-Jack I, Gill CM, Aquilanti E, Bertalan M, Kaplan A, D’Andrea MR, Chukwueke U, Ippen FM et al (2020) Genomic characterization of human brain metastases identifies drivers of metastatic lung adenocarcinoma. Nat Genet 52:371–377. 10.1038/s41588-020-0592-7 PMC7136154 32203465

[60] Wang H, Ou Q, Li D, Qin T, Bao H, Hou X, Wang K, Wang F, Deng Q, Liang J, Zheng W et al (2019) Genes associated with increased brain metastasis risk in non-small cell lung cancer: comprehensive genomic profiling of 61 resected brain metastases versus primary non-small cell lung cancer (Guangdong Association Study of Thoracic Oncology 1036). Cancer 125:3535–3544. 10.1002/cncr.32372 31287555

[61] Lee WC, Reuben A, Hu X, McGranahan N, Chen R, Jalali A, Negrao MV, Hubert SM, Tang C, Wu CC, Lucas AS et al (2020) Multiomics profiling of primary lung cancers and distant metastases reveals immunosuppression as a common characteristic of tumor cells with metastatic plasticity. Genome Biol 21:271. 10.1186/s13059-020-02175-0 PMC7640699 33148332

[62] Andrews LJ, Thornton ZA, Saleh R, Dawson S, Short SC, Daly R, Higgins JPT, Davies P, Kurian KM (2023) Genomic landscape and actionable mutations of brain metastases derived from non-small cell lung cancer: a systematic review. Neuro-Oncol Adv 5:vdad145. 10.1093/noajnl/vdad145 PMC10734675 38130901

[63] Philpott C, Tovell H, Frayling IM, Cooper DN, Upadhyaya M (2017) The NF1 somatic mutational landscape in sporadic human cancers. Hum Genomics 11:13. 10.1186/s40246-017-0109-3 PMC5480124 28637487

[64] Harvey LA (2018) Nearly significant if only…. Spinal Cord 56:1017–1017. 10.1038/s41393-018-0214-8 30397323

[65] Amrhein V, Greenland S, McShane B (2019) Scientists rise up against statistical significance. Nature 567:305–307. 10.1038/d41586-019-00857-9 30894741

[66] Ioannidis JP (2005) Why most published research findings are false. PLoS Med 2:e124. 10.1371/journal.pmed.0020124 PMC1182327 16060722

[67] Cancer Genome Atlas Research Network (2014) Comprehensive molecular profiling of lung adenocarcinoma. Nature 511:543–550. 10.1038/nature13385 PMC4231481 25079552

[68] Zuccato JA, Mamatjan Y, Nassiri F, Ajisebutu A, Liu JC, Muazzam A, Singh O, Zhang W, Voisin M, Mirhadi S, Suppiah S et al (2025) Prediction of brain metastasis development with DNA methylation signatures. Nat Med 31:116–125. 10.1038/s41591-024-03286-y PMC11750707 39379704

[69] Tsakonas G, Koulouris A, Kazmierczak D, Botling J, Ortiz-Villalon C, Nord H, Lindskog M, Sandelin M, Micke P, Hydbring P, Ekman S (2022) Matched analyses of brain metastases versus primary non-small cell lung cancer reveal a unique microRNA signature. Int J Mol Sci. 10.3390/ijms24010193 PMC9820685 36613642

[70] Tsakonas G, Lewensohn R, Botling J, Ortiz-Villalon C, Micke P, Friesland S, Nord H, Lindskog M, Sandelin M, Hydbring P, Ekman S (2020) An immune gene expression signature distinguishes central nervous system metastases from primary tumours in non-small-cell lung cancer. Eur J Cancer 132:24–34. 10.1016/j.ejca.2020.03.014 32325417

[71] WHO Classification of Tumours Editorial Board (2021) Thoracic tumours (Internet). International Agency for Research on Cancer, Lyon

[72] Norwegian Institute of Public Health; Cancer Registry of Norway (2015) Årsrapport 2013–2014: Nasjonalt kvalitetsregister for lungekreft. https://www.fhi.no/globalassets/publikasjoner-og-rapporter/arsrapporter/publisert-2015/aarsrapport_2014_lungekreft.pdf. Accessed 02 Oct 2026

[73] Norwegian Institute of Public Health (2023) Utbredelse av røyking i Norge. https://www.fhi.no/le/royking/tobakkinorge/bruk-av-tobakk/utbredelse-av-royking-i-norge/. Accessed 02 Oct 2026

