# Supplementary material for: Molecular, histopathological, and clinical risk factors for overall metastatic disease and brain metastases in surgically resected stage I–III lung cancer
Source: Clin Exp Metastasis. 2026 Oct 23;43(6):61. doi: 10.1007/s10585-026-10444-8 (PMC13694483; doi:10.1007/s10585-026-10444-8)
Supplement: Supplementary file 1 [file 10585_2026_10444_MOESM1_ESM.pdf]

Supplementary Information

Article name: Molecular, histopathological, and clinical risk factors for overall metastatic disease and brain metastases in surgically resected stage I-III lung cancer

Journal name: Clinical and Experimental Metastasis

Anne Pernille Harlem Dyrbekk[1,2,3], Pål Suhrke[2], Marianne Odnakk Ludahl[5], Nermin Zecic[5], Joakim Oliu Moe[6], Inger Johanne Zwicky Eide[3,7], Marius Lund-Iversen[4] and Odd Terje Brustugun[1,3,7]

[1]University of Oslo, NO-0316 Oslo, Norway
[2]Department of Pathology, Vestfold Hospital Trust, NO-3103 Tønsberg, Norway
[3]Department of Cancer Genetics, Institute for Cancer Research, The Norwegian Radium Hospital, NO-0310 Oslo, Norway
[4]Department of Pathology, Oslo University Hospital, The Norwegian Radium Hospital, NO-0310 Oslo, Norway
[5]Department of Microbiology, Division for gene-technology, Vestfold Hospital Trust, NO-3103 Tønsberg, Norway
[6]Department of Gastroenterology, Vestfold Hospital Trust, NO-3103 Tønsberg, Norway
[7]Department of Oncology, Vestre Viken Hospital Trust, NO-3004 Drammen, Norway

Corresponding author:
Anne Pernille Harlem Dyrbekk
Email: anndyr@siv.no

Content

Extraction DNA and RNA ... 2
Quality requirement Oncomine ... 2
Table A1 Active smoking versus former smoking ... 3
Table A2. Risk of metastasis stratified by TNM-stage ... 4
Table A3. Time to metastasis and time from metastasis to death in the whole cohort ... 5
Table A4. Risk of metastasis in different p-stages in LUAD and LUSC ... 7
Table A5. P-stage. Time to metastasis and time from metastasis to death in LUAD and LUSC ... 8
Table A6. Demographic, histopathological characteristics, risk of extracranial metastasis ... 9
Table A7. Clinicopathological characteristics early versus late metastasis ... 11
Table A8. Time to lung cancer related death and all cause death in the whole cohort and in non-metastatic subgroup ... 14
Table A9. Clinicopathological characteristics LUAD and LUSC with NGS-results versus not screened with NGS ... 16
Table A10a. NGS testing primary tumour, all cases ... 17
Table A10b. NGS testing primary tumour in LUAD and LUSC in different p-stages and metastatic pattern ... 19
Table A11. Mutations. Time to metastasis and time from metastasis to death in LUAD and LUSC. KRAS and EGFR excluded. ... 22
Table A12a. EGFR PCR or NGS adenocarcinoma ... 24
Table A12b. EGFR PCR or NGS adenocarcinoma stratified by p-stage and later metastasis ... 25

Table A13. Subtypes EGFR mutations 26
Table A14a. KRAS PCR or NGS adenocarcinoma 26
Table A14b. KRAS PCR or NGS adenocarcinoma stratified by p-stage and later metastasis 28
Table A15. Subtypes KRAS mutations 28
Figure A1. Kaplan-Meier survival estimates metastasis by risk of brain metastasis by clinicopathological parameters 29
Figure A2. Kaplan-Meier survival estimates metastasis by risk of brain metastasis by molecular parameters 32

Extraction DNA and RNA

Isolation from fresh-frozen tissue: AllPrep DNA/RNA/miRNA Universal Kit (Qiagen, 80224) on the QIAcube (automated, spin-column-based nucleic acid extraction from Qiagen).

Concentration measurement: Nanodrop (Thermo Fisher Scientific) and Qubit Fluorometer (Thermo Fisher Scientific).

RNA-quality: Agilent 2100 Bioanalyzer (Agilent Technologies)

Extraction from FFPE-blocks:

DNA: MagLead12gC (Biosystem, A1120)

RNA: Quick RNA FFPE kit (Zymo Research, R1008) for RNA.

Quality requirement Oncomine

**TORRENT SUITE**	
% Loading	At least 60%
% Polyclonality	45% or less
Low Quality	<20% for DNA, higher for RNA because of degraded material
Total Reads	DNA+RNA: 45 000 000-75 000 000
Median reading length for DNA	110-115 bp
Medium reading length for RNA	65-110 bp
**DNA**	
Mapped Reads	3 000 000- 6 000 000
% reads on target	>90%
Mean depth	>1200
Uniformity	>95%, for degraded material: 85-90%
Base coverage depth	>800 bp
Number of amplicons	3781
**RNA**	
Mapped Reads	500 000-1 500 000 (minimum 40 000)
**ION REPORTER**	
**DNA**	
Mutation (QC)	Phred QUAL Score >20<br>Coverage>1000
CNV	Copy number ≥8<br>MAPD <0,5
**RNA**	

Total Mapped Fusion Panel Reads	>500 000
Expression control counts	Read counts >15
Expression control detected	In total 6 (3 in each pool, should see at least 2-3.)
Fusion	Read counts >1000
Skipping and deletions	Read counts >1000

Table A1 Active smoking versus former smoking.

	**Active n=416**		**Former n=432**	
**Time since smoking cessation**			<0,5 years: 36<br>0,5-1 year: 30<br>1-2 Years: 51<br>2-4 Years: 51<br>>4 years: 264	
**Metastasis**	149	Brain: 47	171	Brain: 56
		Other:102		Other: 115
**Non-metastasis**	267		261	
**Time to metastasis***, year (median/mean)**	1.5 /2.1		1.5/2.2	
**Time to death***, year (median/mean)**	5.2/5.1		5.6/5.7	

Table A1. No significant association with active smoking and developing metastasis, brain metastasis and early metastasis compared with former smoking. With survival analysis (cox regression and log rank) there were no significant association with active smoking versus former smoking and metastasis, lung cancer related death and overall survival. *** Time from surgery to event among patients who experienced the event during follow-up.

Table A2. Risk of metastasis stratified by TNM-stage

	All	Metastatic	Non-metastatic	Risk of metastasis.<br><br>Total: All cases<br>HR, 95% CI, p-value	Brain-metastasis	Brain-metastasis versus all others<br>HR, 95% CI, p-value	Extracranial metastasis	Risk of extracranial metastasis versus all others<br>HR, 95% CI, p-value
**T-stage**								
0	2 (0.2%)		1 (0.2%)					
1a	168 (18.4%)	44 (12.8%)	124 (21.8%)	Ref	14 (12.4%)	Ref	30 (13.0%)	Ref
1b	150 (16.5%)	45 (13.1%)	105 (18.5%)	n.s.	10 (8.9%)	n.s.	35 (15.2%)	n.s.
1c	1 (0.1%)	-	1 (0.2%)	-	-	-	-	-
2a	332 (36.4%)	133 (38.7%)	199 (35.0%)	1.72, 1.22-2.42, p=0.002	40 (35.4%)	n.s.	93 (40.3%)	1.78, 1.18-2.69, p=0.006
2b	96 (10.5%)	40 (11.6%)	56 (9.9%)	1.88, 1.22-2.88, p=0.004	17 (15.0%)	2.37, 1.11-4.66, p=0.037	23 (10.0%)	1.61, 0.94-2.78, p=0.085
3	132 (14.5%)	70 (20.4%)	62 (10.9%)	2.99, 2.05-4.36, p<0.001	32 (26.6%)	3.23, 1.64-6.40, p=0.001	40 (17.3%)	2.57, 1.60-4.12, p<0.001
4	30 (3.3%)	12 (3.5%)	18 (3.2%)	2.00, 1.06-3.80, p=0.033	2 (1.8%)	n.s.	10 (4.3%)	2.53, 1.24-5.19, p=0.011
Missing	2 (0.2%)		2 (0.4%)					
**N-stage**								
0	660 (72.4%)	211 (61.3%)	449 (79.1%)	Ref	60 (53.1%)	Ref	151 (65.4%)	Ref
1	179 (19.6%)	92 (26.7%)	87 (15.3%)	2.08, 1.62-2.65, <0.001	33 (29.2%)	2.26, 1.42-3.59, p=0.001	59 (25.5%)	1.90, 1.40-2.56, p<0.001
2	71 (7.8%)	41 (11.9%)	30 (5.3%)	2.60, 1.86-3.63, p<0.001	20 (17.7%)	3.92, 2.19-7.01, p=<0.001	21 (9.1%)	1.92, 1.22-3.03, p=0.005
Missing	2 (0.2%)		2 (0.4%)					

Table A2: TNM stage and estimates of associations from univariate cox regression models. HR: Hazard ratio. 95% CI: 95% confidence interval. Ref: reference group. n.s.: Not significant (p>0.1

Table A3. Time to metastasis and time from metastasis to death in the whole cohort

	Time to met. (years)***	HR*/ Chi²**	Time to BM (years)***	HR*/ Chi²**	Time from BM to death (years)***	HR*/ Chi²**	Time to ECM (years)***	HR*/ Chi²**	Time from ECM to death (years)***	HR*/ Chi²**
**All**	1.48		1.01		0.73		1.90		0.69	
**Sex**										
Female	1.63	Ref.	1.34	Ref	0.97	Ref.	2.17	Ref.	0.69	Ref.
Male	1.39	n.s./n.s.	0.86	n.s.	0.57	n.s./n.s.	1.81	n.s./n.s.	0.69	1.37, 1.03-1.84, p=0.032/4.67, p=0.031
**Agegroup**										
<50	0.47	Ref.	0.47	Ref	3.55	Ref.	0.56	Ref.	1.13	Ref.
≥50	1.52	n.s.[1]/n.s.	1.03	n.s.[1]	0.73	n.s.*/n.s.	1.93	n.s.	0.68	n.s./n.s.
<60	1.40	Ref.	0.93	Ref.	1.15	Ref.	2.05	Ref.	0.95	Ref.
≥60	1.50	n.s./n.s.	1.07	0.63, 0.41-0.95, p=0.027/4.95, 0.026	0.62	1.52, 0.96-2.42, P=0.077/ 3.18, p=0.074	1.83	n.s.	0.67	n.s./n.s.
<70	1.64	Ref.	0.97	Ref.	0.77	Ref.	2.13	Ref.	0.69	Ref.
≥70	1.36	n.s.	1.04	0.67, 0.44-1.01, p=0.059/ 3.62, p=0.06	0.57	n.s./n.s.	1.48	n.s./n.s.	0.62	1.49, 1.11-2.00, p=0.007 / n.s.
**Smoking**										
Never smoking	1.29	Ref.	0.72	Ref.	3.16	Ref.	2.13	Ref.	1.18	Ref.

Former/current smoking	1.49	n.s./n.s.	1.02	n.s./n.s.	0.62	2.04, 0.98-4.23, p=0.056[1]/ 3.83, p=0.050	1.83	n.s.	0.67	n.s./n.s.
**Histopathological type**										
LUSC	1.39	Ref.	0.59	Ref.	0.40	Ref.	1.48	Ref.	0.58	Ref.
LUAD	1.69	1.33, 1.04-1.70, p=0.025/ 5.02, 0.025	1.04	3.62, 2.02-6.50, p<0.001/ 21.37, p<0.001	0.88	n.s./n.s.	2.26	n.s./n.s.	0.84	0.70, 0.51-0.95, p=0.024/5.12, p=0.024
ADSQC	1.34	2.89, 1.54-5.41, p=0.001/ 11.64, <0.001	0.83	6.71, 2.19-20.58, p=0.001/13.29, p<0.001	0.64	n.s./n.s.	1.60	2.22, 1.02-4.83, p=0.043/ 4.07, p=0.04	0.50	n.s./n.s.
Salivary gland-type carcinoma	2.39	n.s.	-	-/-	-	-	2.39	n.s./n.s.	2.40	n.s./n.s.
LCC	0.67	2.42, 1.38-4.26, p=0.002/ 9.85, 0.002	0.21	3.46, 0.98-12.13, p=0.053/3.77, p=0.052	0.27	n.s./n.s.	0.85	2.25, 1.20-4.24, p=0.012/ 6.60, p=0.010	0.63	n.s./n.s.
LCNEC	1.47	3.46, 1.60-7.47, p=0.002/ 10.33, 0.001	2.07	8.85, 2.52-31.13, p=0.001/29.47, p<0.001	1.06	n.s./n.s.	1.14	2.48, 0.90-6.79, p=0.078/ 2.80, p=0.09	0.50	n.s./n.s.
Mixed	0.69	12.88, 1.78-93.32, p=0.011/ 10.37, 0.001	0.70	61.84, 7.94-481.57, p<0.001	-	-	-	-	-	-
Other	-	n.s./n.s.	-	-	-	-	-	-	-	-

SCLC	1.98	n.s./n.s.	1.73	6.88, 2.24-21.09, p=0.001/14.42, p<0.001	1.43	n.s./n.s.	5.06	n.s./n.s.	0.56	n.s./n.s.
Undifferentiated carcinoma	0.53	5.80, 1.83-18.38, p=0.003/ 10.55, 0.001	-	-	-	-	0.53	7.77, 2.44-24.78, p=0.001/ 14.29, p<0.001	0.90	n.s/n.s.

Table A3. Median time (years) to brain metastasis or extracranial metastasis, and median time from metastasis to all cause death. Met.: Metastasis. LUAD: Lung adenocarcinoma LUSC: Lung squamous cell carcinoma ADSQC: Adenosquamous carcinoma LCC: Large cell carcinoma SCLC: Small cell carcinoma LCNEC: Large cell neuroendocrine carcinoma. ***Median time to event among patients who experienced the event during follow-up. * Cox regression. HR, 95% CI, p-value ** Log Rank Chi2, Pr>chi2 [1] Schoenfeld residuals test (χ^2, P>|χ^2|); P<0.05 indicates PH violation.

Table A4. Risk of metastasis in different p-stages in LUAD and LUSC

	LUAD							LUSC						
**P-stage**	**Total**	**Met.**	**No met.**	**Risk of met.** Total: All LUAD cases. HR, 95% CI, p-value	**BM**	**ECM**	**Risk of BM** Total: All LUAD cases, HR, 95% CI, p-value	**Total**	**Met.**	**No met.**	**Risk of met.** Total: All LUSC cases. HR, 95% CI, p-value	**BM**	**ECM**	**Risk of BM** Total: All LUSC cases, HR, 95% CI, p-value
**Ia**	165	36	129	ref	13	23	ref	74	18	56	ref	-	18	-
**Ib**	147	56	91	1.98, 1.30-3.00, p=0.001	21	35	2.00, 1.00-4.00, p=0.049	79	21	58	n.s.	-	21	-
**IIa**	91	43	48	2-76, 1.77-4.30, p<0.001	14	29	2.39, 1.12-5.09, p=0.024	62	16	46	n.s.	4	12	Too few
**IIb**	52	28	24	3.41, 2.08-5.59, p<0.001	13	15	4.20, 1.94-9.06, p<0.001	35	12	23	n.s.	1	11	Too few

**IIIa**	76	47	29	4.57, 2.95-7.06, p<0.001	23	24	5.57, 2.82-11.02, p<0.001	48	21	27	2.43, 1.29-4.57, p=0.006	8	13	-
**IIIb**	2	2	-	168.51, 36.21-784.26, p<0.001	1	1	121.16, 14.28-1028.29, p<0.001	0	-	-	-	-	-	-
**Missing**								2		2				
**Total**	533	212	321		85	127		300	88	212		13	75	

Table A4. HR: Hazard ratio. 95% CI: 95% confidence interval. Ref: reference group. n.s.: Not significant (p>0.1)

Table A5. P-stage. Time to metastasis and time from metastasis to death in LUAD and LUSC

		**Time to met. (years)[1]**	**HR*/ Chi^2****	**Time to BM (years)[1]**	**HR*/ log rank**	**Time from BM to death (years)[1]**	**HR*/ Chi^2****	**Time to ECM (years)[1]**	**HR*/ Chi^2****	**Time from ECM to death (years)[1]**	**HR*/ log rank****
**Ia**	**LUAD**	2.37	Ref.	1.71	Ref.	0.30	Ref.	3.02	Ref.	0.85	Ref.
**Ib**	**LUAD**	1.59	1.98, 1.30-3.00, p=0.001/10.53, p=0.001	1.01	2.00, 1.00-4.00, p=0.049/ 4.10, p=0.043	0.90	0.42, 0.20-0.87, p=0.020 / 4.71, p=0.030	1.96	1.96, 0.16-3.32, p=0.012/ 6.43, p=0.011	1.18	n.s./n.s.
**IIa**	**LUAD**	1.69	2.76, 1.77-4.30, p<0.001/ 22.45, p<0.001	1.07	2.39, 1.12-5.09, p=0.024/ 5.89, p=0.015	1.23	0.43, 0.20-0.94, p=0.034 / 5.18, p=0.023	2.18	2.99, 1.73-5.16, p<0.001/ 16.75, p<0.001	0.87	n.s./n.s.

**IIb**	**LUAD**	1.39	3.41, 2.08-5.59, p<0.001/ 27.29, p<0.001	0.99	4.20, 1.94-9.06, p<0.001/ 16.72, p<0.001	1.18	n.s./ n.s.	2.66	2.94, 1.53-5.64, p=0.001/ 11.50, p<0.001	0.91	n.s./n.s.
**IIIa**	**LUAD**	1.53	4.57, 2.95-7.06, p<0.001/ 56.09, p<0.001	0.77	5.57, 2.82-11.02, p<0.001/ 30.25, p<0.001	0.82	0.38, 0.18-0.78, p=0.008 / 5.48, 0.019	2.07	3.92, 2.20-6.96, p<0.001/ 26.14, p<0.001	0.63	n.s./n.s.
**IIIb**	**LUAD**	0.17	168.51, 36.20-784.26, p<0.001/ 220.00, p<0.001	0.23	121.16, 14.28-1028.29, p<0.001/ 164.00, p<0.001	0.27	n.s./ n.s.	0.10	316.49, 31.19-3211.92, p<0.001/ 82.50, p<0.001	0.06	-/ 23.00, p<0.001
**Ia**	**LUSC**	2.01	Ref.	-	Ref.	-	Ref.	2.01	Ref.	0.72	Ref.
**Ib**	**LUSC**	2.07	n.s./n.s.	-	-	-	-	2.07	n.s./n.s.	0.58	n.s./n.s.
**IIa**	**LUSC**	1.24	n.s./n.s.	0.71	-	0.62	-	1.57	n.s./n.s.	0.30	n.s./n.s.
**IIb**	**LUSC**	0.74	n.s./n.s.	0.76	n.s.	0.37	n.s.	0.73	n.s./n.s.	0.44	n.s./n.s.
**IIIa**	**LUSC**	0.98	HR 2.43, 1.29-4.57, p=0.006/ 7.79, p=0.005	0.50	n.s.	0.33	n.s.	1.03	n.s./n.s.	0.88	n.s./n.s.
**IIIb**	**LUSC**	-	-	-	-	-	-	-		-	-

LUAD: Lung adenocarcinoma. LUSC: Lung squamous cell carcinoma. [1]Median time to event among patients who experienced the event during follow-up* Cox regression. HR, 95% CI, p-value ** Log Rank Chi2, Pr>chi2

Table A6. Demographic, histopathological characteristics, risk of extracranial metastasis

	**All (% of total)**	**Metastatic**	**Non-metastatic**	**ECM**	**Risk of ECM**<br><br>**Total: All cases (n=912)**<br>**HR, 95% CI, p-value**
**Number**	912 (100%)	344	568	231	
**Sex**					
Female	442 (48.5%)	167 (48.6%)	275 (48.4%)	107 (46.3%)	ref
Male	470 (51.5%)	177 (51.5%)	293 (51.6%)	124 (53.7%)	n.s.

**Age at surgery (median)**	67.6	67.3	67.8	68.2	n.s.
**Agegroup 60**					
< 60 years	171 (18.8%)	71 (20.6%)	100 (17.6%)	40 (17.3%)	
≥ 60 years	741 (81.3%)	273 (79.4%)	468 (82.4%)	191 (82.7%)	n.s.
**Smoking**					
Never smoker	64 (7.0%)	24 (7.0%)	40 (7.0%)	14 (6.1%)	ref
Former/current smoker	848 (93.1%)	320 (93.0%)	528 (93.0%)	217 (93.9%)	n.s.
**Pack year (median)**	31.5	31.8	30.8	36.0	1.01, 1.00-1.01, p=0.007
**Histology**					
LUSC	300 (32.9%)	88 (25.6%)	212 (37.3%)	75 (32.5%)	ref
LUAD	533 (58.4%)	212 (61.6%)	321 (56.5%)	127 (55.0%)	n.s.
LCC	26 (2.9%)	14 (4.1%)	12 (2.1%)	11 (4.8%)	2.25, 1.20-4.24, p=0.012
ADSQC	16 (1.8%)	11 (3.1%)	5 (0.9%)	7 (3.0%)	2.22, 1.02-4.83, p=0.043
Small cell carcinoma	13 (1.4%)	6 (1.7%)	7 (1.2%)	2 (0.9%)	n.s.
LCNEC	10 (1.1%)	7 (2.0%)	3 (0.5%)	4 (1.7%)	2.48, 0.90-6.79, p=0.078
Salivary gland-type carcinoma	5 (0.6%)	2 (0.6%)	3 (0.5%)	2 (0.9%)	n.s.
Undifferentiated carcinoma	4 (0.4%)	3 (0.9%)	1 (0.2%)	3 (1.3%)	7.77, 2.43-24.78, p=0.001
Other	3 (0.3%)	-	3 (0.5%)	-	-
Mixed	2 (0.2%)	1 (0.3%)	1 (0.2%)	-	-
**P-stage**					
Ia	259 (28.4%)	63 (18.3%)	196 (34.5)	47 (20.4%)	ref

Ib	242 (26.5%)	84 (24.4%)	158 (27.8%)	60 (26.0%)	1.49, 0.97-2.29, p=0.071
IIa	177 (19.4%)	76 (22.1%)	101 (17.8%)	52 (22.5%)	1.88, 1.19-2.95, p=0.006
IIb	95 (10.4%)	46 (13.4%)	49 (8.6%)	32 (13.9%)	2.29, 1.35-3.89, p=0.002
IIIa	134 (14.7%)	73 (21.2%)	61 (10.7%)	39 (16.9%)	1.85, 1.14-3.02, p=0.013
IIIb	3 (0.3%)	2 (0.6%)	1 (0.2%)	1 (0.4%)	n.s.
Missing	2 (0.2%)	-	2 (0.4%)	-	

Table A6: Demographic and histopathological characteristics and estimates of associations from univivariate cox regression models. n.s.: Not significant (p>0.1). HR: Hazard ratio. 95% CI: 95% confidence interval. Ref: Reference group Met. Metastasis. BM: Brain metastasis ECM: Extracranial metastasis LUSC: Lung squamous cell carcinoma LUAD: Lung adenocarcinoma LCC: Large cell carcinoma ADSQC: Adenosquamous carcinoma LCNEC: Large cell neuroendocrine carcinoma

Table A7. Clinicopathological characteristics early versus late metastasis

**Parameters**	**Early metastasis (<0,5year) (%of total)**	**Late metastasis (>/= 0,5years) (%of total)**	**OR early metastasis vs late metastasis, 95%CI, p-value**	**OR early BM vs late BM, 95%CI, p-value**
**Number**	59 (100%)	285 (100%)		
**Metastatic site**				
ECM	33 (55.9%)	198 (69.5%)	Ref.	
BM	26 (44.1%)	87 (30.5%)	1.80, 1.01-3.18, p=0.046	-
**Sex**				
Female	23 (39.0%)	144 (50.5%)	ref	ref.
Male	36 (61.0%)	141 (49.5%)	n.s.*	2.68, 1.07-6.67, p=0.035
**Age (median)**	67.7	67.2	n.s.	n.s.
**Agegr. </> 50 years**				

<50 years	7 (11.9%)	5 (1.75%)	ref	Ref.
>50 years	52 (88.1%)	280 (98.3%)	0.13, 0.04-0.43, p=0.001	0.06, 0.01-.0.60, p=0.016
**Agegr. </> 60 years**				
<60 years	15 (25.4%)	56 (19.7%)	ref	ref.
>/= 60 years	44 (74.6%)	229 (80.4%)	n.s.	n.s.
**Smoking**				
Never smoker	7 (11.9%)	17 (6.0%)	ref	ref
Form./curr. smoker	52 (88.1%)	268 (94.0%)	n.s.	n.s.
**PY (median)**	30.1	32.3	n.s.	n.s.
**Histology**				
LUSC	16 (27.1%)	72 (25.3%)	ref	ref.
LUAD	36 (61.0%)	176 (62.8%)	n.s.	n.s.
LCC	12 (10.2%)	8 (2.8%)	3.38, 1.03-11.08, p=0.045	-
ADSQC	-	11 (3.9%)	-	-
SCLC	-	6 (2.11%)	-	-
LCNEC	-	7 (2.5%)	-	-
Saliv. gl carc.	-	2 (0.7%)	-	-
Undiff. carc.	1 (1.7%)	2 (0.7%)	n.s.	-
Other	-	-	-	-
Mixed	-	1 (0.4%)	-	-
**P-stadium**				
Ia	7 (11.9%)	56 (19.7%)	ref	ref.
Ib	7 (11.9%)	77 (27.0%)	n.s.	0.29, 0.08-1.02, p=0.054

IIa	13 (22.0%)	63 (22.0%)	n.s.	n.s.
IIb	10 (17.0%)	36 (12.6%)	n.s.	n.s.
IIIa	20 (33.9%)	53 (18.6%)	3.02, 1.18-7.72, p=0.021	-
IIIb	2 (3.4%)	-	-	-
**T-stage**				
0	-	-		
1a	5 (8.5%)	39 (13.7%)	ref	ref.
1b	5 (8.5%)	40 (14.04%)	n.s.	n.s.
1c	-	-	-	-
2a	14 (23.7%)	119 (41.8%)	n.s.	n.s.
2b	7 (11.9%)	33 (11.6%)	n.s.	n.s.
3	26 (44.1%)	44 (15.4%)	4.61, 1.61-13.2, p=0.004	n.s.
4	2 (3.4%)	10 (3.5%)	n.s.	-
Missing	-	-		
**N-stage**				
0	28 (47.5%)	183 (64.2%)	Ref	ref.
1	19 (32.2%)	73 (25.6%)	n.s.	4.33, 1.50-12.49, p=0.007
2	12 (20.3%)	29 (10.2%)	2.7, 1.2-5.9, p=0.013	4.08, 1.22-13.68, p=0.023

Table A7. Clinicopathological characteristics early versus late metastasis. BM: Brain metastasis ECM: Extracranial metastasis N.s.: Not significant p>0.100 Agegr.: Agegroup Form./curr. Smoker: Former/current smoker PY: Pack year LUAD: Lung adenocarcinoma LUSC: Lung squamous cell carcinoma ADSQC: Adenosquamous carcinoma LCC: Large cell carcinoma SCLC: Small cell carcinoma LCNEC: Large cell neuroendocrine carcinoma Saliv. gl carc: Salivary gland-type carcinoma Undiff. carc.: Undifferentiated carcinoma. *Sex become significant in a multivariable logistic regression model adjusting for age and smoking (OR 1.89, 1.04-3.44, p=0.038).

Table A8. Time to lung cancer related death and all cause death in the whole cohort and in non-metastatic subgroup

	**Median time to lung cancer specific death (years)*** **	**Median time to lung cancer specific death (years) if BM occurs*** **	**Median time to death (years)*** **	**Median time to death (years), non-metastatic patients*** **
All	2.43	2.45	2.94	5.66
**Sex**				
Female	2.76	2.73	3.08	5.68
Male	2.16	1.98	2.75	5.62
**Agegroup**				
<50	1.97	7.03	1.97	8.96
≥50	2.44	2.36	2.96	5.62
**Agegroup**				
<60	2.93	3.52	3.23	7.16
≥60	2.42	2.21	2.88	5.46
**Smoking**				
Never smoking	3.15	3.16	3.66	5.69
Former/current smoking	2.38	2.21	2.86	5.65
**Histopathological type**				
LUSC	2.08	1.13	2.62	5.38
LUAD	2.78	2.70	3.31	6.06
ADSQC	1.96	1.75	2.42	5.66

Salivary gland-type carcinoma	3.09	-	3.09	7.72
LCC	1.39	0.51	1.78	5.75
LCNEC	1.95	3.52	1.95	3.69
Mixed	0.04	-	0.04	0.04
Other	0.19	-	5.10	5.10
SCLC	3.49	2.92	3.49	5.43
Undifferentiated carcinoma	0.38	-	2.04	2.04

Table A8. Median time (years) to metastasis, lung cancer related death and all cause death. LUAD: Lung adenocarcinoma LUSC: Lung squamous cell carcinoma ADSQC: Adenosquamous carcinoma LCC: Large cell carcinoma SCLC: Small cell carcinoma LCNEC: Large cell neuroendocrine carcinoma. *** Time to event among patients who experienced the event during follow-up.

Table A9. Clinicopathological characteristics LUAD and LUSC with NGS-results versus not screened with NGS

		NGS+ (LUAD and LUSC)	NGS– (LUAD and LUSC)
Number total		113	720
Sex	Female	50 (44.3%)	355 (49.3%)
	Male	63 (55.8%)	365 (50.7%)
Age at surgery (median/mean)		67.1/66.5	67.7/67.1
Agegroup </> 50	<50 years	4 (3.5%)	25 (3.4%)
	≥50 years	109 (96.5%)	702 (96.6%)
Agegroup </> 60	<60 years	21 (18.6%)	129 (17.9%)
	>60years	92 (81.4%)	591 (82.1%)
Smoking	Never smoker	13 (11.5%)	48 (6.7%)
	Former/current smoker	100 (88.5%)	672 (93.3%7
Pack year (median/mean)		31.5 (32.8)	31.4 (32.7)
Histology, compared to all other groups combined	Adenocarcinoma	69 (61.1%)	464 (64.4%)
	Squamous cell carcinom	44 (38.9%)	256 (35.6%)
P-stadium	Ia	32 (28.3%)	207 (28.8%)
	Ib	26 (23.0%)	200 (27.8%)
	IIa	20 (17.7%)	133 (18.5%)
	IIb	16 (14.2%)	71 (9.9%)
	IIIa	19 (16.8%)	105 (14.6%)
	IIIb		2 (0.3%)
	Missing		2 (0.3%)
T-stage	0		1 (0.1%)
	1a	20 (17.7%)	137 (19.0%)
	1b	20 (17.7%)	112 (15.6%)
	1c		1 (0.1%)
	2a	39 (34.5%)	264 (36.7%)
	2b	10 (8.9%)	79 (11.0%)
	3	21 (18.6%)	100 (13.9%)
	4	3 (2.7%)	24 (3.3%)
	Missing		2 (0.3%)
N-stage	0	79 (69.9%)	531 (73.8%)
	1	24 (21.2%)	132 (18.3%)
	2	10 (8.9%)	55 (7.6%)
	Missing		2 (0.3%)

Table A10a. NGS testing primary tumour, all cases.

	All	
**Histopathology**	**Total tested**	**Mutation**
**All**	**127**	
**LUAD**	69/533	Mutation:<br>TP53 (n= 34, 49.3%)<br>KRAS (n= 23, 33.3%)<br>EGFR (n= 11, 15.9%)<br>RB1 (n= 6, 8.7%)<br>SETD2 (n= 4, 5.8%)<br>PTCH1, STK11 (n=3, 4.3%)<br>ATRX, BRCA2, CREBBP, MAP2K1, NOTCH3, PIK3R1 (n= 2, 2.9%)<br>ARID1A, BRCA1, CBL, CDK4, IDH1, NF1, NOTCH1, NRAS, PALB2, RAD50, STAT3, TSC2, U2AF1 (n=1, 1.4%)<br>Negative (n=5, 7.2%)<br><br>CNV:<br>AKT2, AKT3, CCND1, CDK6, EGFR, FGF3, FGFR1, FGF19, KIT, MYC, PDGFRA, PIK3CA, RICTOR, TERT (n=1, 1.4%)<br>Negative (n=61, 88.4%)<br><br>Fusion:<br>ALK, RET, MET exon 14 skipping (n=1, 1.4%)<br>ROS1 (n=3, 4.3%)<br>Failed (n=2, 2.9%)<br>Negative (n=61, 88.4%)
**LUSC**	44/300	Mutation:<br>TP53 (n=24, 54.5%)<br>NFE2L2 (n=5, 11.4%)<br>FBXW7, PTEN, RAD50, PIK3CA (n= 3, 6.8%)<br>ARID1A, SETD2 (n=2, 4.6%)<br>ATRX, CDKN2A, ESR1, FANCD2, MET, NOTCH1, PTCH1, KRAS, SLX4, STK11, TSC1 (n=1, 2.3%)<br>Negative (n=12, 27.3%)

		CNV:<br>FGFR1 (n=1, 2.3%)<br>Failed (n=2, 4.6%)<br>Negative (n=41, 93.2%)<br><br>Fusion:<br>FGFR3 (n=1, 2.3%)<br>Failed (n=1, 2.3%)<br>Negative (n=42, 95.5%)
**ADSQC**	1/16	Mutation and CNV:<br>Failed (n=1, 100%)<br><br>Fusion:<br>Negative (n=1, 100%)
**LCC**	3/26	Mutation:<br>PTEN (n=1, 33.3%)<br>TP53 (n=1, 33.3%)<br>Failed (n=1, 33.3%)<br>Negative (n=1, 33.3%)<br><br>CNV:<br>Failed (n=1, 33.3%)<br>Negative (n=2, 66.7%)<br>Fusion:<br>Failed (n=1, 33.0%)<br>Negative (n=2, 66.7%)
**SCLC**	0/13	-
**LCNEC**	5/10	Mutation:<br>TP53 (n=3, 60.0%)<br>ARID1A, ATRX, MAP2K1, NF1, PALB2, SMARCA4, STK11 (n=1, 20.0%)<br>Negative (n=1, 20.0%)<br>CNV:<br>MYCL (n=1, 20.0%)<br>PDGFRA (n=1, 20.0%)<br>Negative (n=4, 80.0%)<br><br>Fusion:<br>Negative (n=4, 80.0%)

**Salivary gland-type carcinoma**	4/5	Mutation:<br>ERBB2, PTCH1, TP53 (n=1, 25.0%)<br>Negative (n=2, 50.0%)<br><br>CNV:<br>CCNE1 (n=1, 25.0%)<br>AKT2 (n=1, 25.0%)<br>Negative (n=3, 75.0%)<br><br>Fusion:<br>MYB (n=1, 25.0%)<br>Negative (n=3, 75.0%)
**Undifferentiated carcinoma**	0/4	-
**Mixed**	1/2	Mutation, CNV and fusion:<br>Negative (n=1, 100%)
**Other**	0/3	-

Table A10a. NGS testing primary tumour, stratified by later metastasis status: Cases that did not develop metastasis, cases that developed brain metastasis and cases that developed extracranial metastasis. LUAD: Lung adenocarcinoma LUSC: Lung squamous cell carcinoma ADSQC: Adenosquamous carcinoma LCC: Large cell carcinoma SCLC: Small cell carcinoma LCNEC: Large cell neuroendocrine carcinoma

Table A10b. NGS testing primary tumour in LUAD and LUSC in different p-stages and metastatic pattern

	**Total/total tested**	**Brain metastasis**		**Extracranial metastasis**		**No metastasis**	
		**Total/ total tested**		**Total/ total tested**		**Total/total tested**	
**LUAD**							
**All stages**	**533/69**	**85/16**		**127/13**		**321/40**	
**p-stage Ia+Ib**	**312/41**	**34/5**	Mutation:<br>TP53 (n=5, 100.0%)<br>MAP2K1 (n=2, 40.0%)<br>EGFR, BRCA2, ATRX1, NF1, PALB2 (n=1, 20.0%)<br><br>CNV:	**58/7**	Mutation:<br>TP53 (n=6, 85.7%)<br>EGFR (n=2, 28.6%)<br>CREBBP, STK11, ERBB2, STAT3 (n=1, 14.3%)<br><br>CNV	**220/29**	Mutation:<br>KRAS (n=11, 37.9%)<br>TP53 (n=9, 31.0%)<br>EGFR (n=5, 17.2%<br>RB1 (n=2, 6.9%)<br>ATRX, PTCH1, STK11, ERBB2, NOTCH3, SETD2, PIK3R1, IDH1, NOTCH1 (n=1, 3.4%)

			TERT, RICTOR, EGFR (n=1, 20.0%)<br>Negative (n=4, 80.0%)<br><br>Fusion:<br>Negative (n=5, 100.0%)		CCND1, FGF19, FGF3 (n=1, 14.3%)<br>Negative (n=6, 85.7%)<br><br>Fusion:<br>Negative (n=7, 100.0%)		Negative (n=4<br><br>CNV:<br>AKT3, CDK6, FGFR1, PDGFRA, PIK3CA, KIT (n=1, 3.4%)<br>Negative (n=25, 86.2%)<br><br>Fusion:<br>ALK, RET (n=1, 3.4%)<br>Negative (n=26, 89.7%)<br>Failed (n=1, 3.4%)
**p-stage IIa+IIb**	**143/20**	**27/8**	Mutation:<br>KRAS (n=6, 75.0%)<br>TP53 (n=3, 37.5%)<br>BRCA2, BRCA1, CBL, CREBBP, RAD50, RB1, U2AF1 (n=1, 12.5%)<br><br>CNV:<br>Negative (n=8, 100.0%)<br><br>Fusion:<br>Negative (n=8, 100.0%)	**44/3**	Mutation:<br>TP53, RAD50, KRAS, STK11 (n=1, 33.3%)<br><br>CNV:<br>AKT2 (n=1, 33.3%)<br>Negative (n=2, 66.7%)<br><br>Fusion:<br>ROS1 (n=1, 33.3%)<br>Negative (n=2, 66.7%)	**72/9**	Mutation:<br>TP53 (n=5, 55.6%)<br>KRAS (n=4, 44.4%)<br>RB1, SETD2 (n=2, 22.2%)<br>EGFR, PTCH1, PIK3R1, ARID1A, CDK4, NRAS, TSC1 (n=1, 11.1%)<br>Negative (n=1, 11.1%)<br><br>CNV:<br>Negative (n=9, 100.0%)<br><br>Fusion:<br>ROS1 (n=2, 22.2%)<br>MET exon 14 skipping (n=1, 11.1%)<br>Negative (n=6, 66.7%)
**p-stage IIIa+IIIb**	**78/8**	**24/3**	Mutation:<br>TP53 (n=2, 66.7%)<br>EGFR (n=2, 66.7%)<br>PTCH1 (n=1, n=33.3%)<br><br>CNV:<br>Negative (n=3, 100.0%)<br><br>Fusion:<br>Negative (n=3, 100.0%)	**25/3**	Mutation:<br>TP53, KRAS, NOTCH3, RB1 (n=1, 33.3%)<br><br>CNV:<br>MYC (n=1, 33.3%)<br>Negative (n=2, 66.7%)<br><br>Fusion:<br>Negative (n=3, 100.0%)	**29/2**	Mutation:<br>TP53 (n=2, 100.0%)<br>SETD2 (n=1, 50.0%)<br><br>CNV:<br>Negative (n=2, 100.0%)<br><br>Fusion:<br>Negative (n=1, 100%)<br>Failed (n=1)
**LUSC**							
**All stages**	**300/44**	**13/4**		**75/13**		**212/27**	
**p-stage Ia+Ib**	**153/17**	**-**		**39/5**	Mutation:<br>TP53 (n=2	**114/12**	Mutation:<br>TP53 (n=5<br>PTEN (n=2

					FBXW7, NFE2L2, ARID1A, ATRX, CDK2NA, RAD50, SETD2 (n=1<br>PIK3CA (n=2<br>Negative (n=1<br><br>CNV:<br>Negative (n=5<br><br>Fusion:<br>Negative (n=5		FBXW7, RAD50, SETD2, KRAS, PTCH1 (n=1<br>Negative (n=5<br><br>CNV:<br>Negative (n=12<br><br>Fusion:<br>Negative (n=12
**p-stage IIa+IIb**	**97/16**	**5/1**	Mutation:<br>Negative (n=1, 100.0%)<br><br>CNV:<br>Negative (n=1, 100.0%)<br><br>Fusion:<br>Negative (n=1, 100.0%)	**23/5**	Mutation:<br>TP53 (n=3<br>NFE2L2 (n=2<br>ESR1, STK11, TSC1 (n=1<br>Negative (n=1<br><br>CNV:<br>FGFR1 (n=1<br>Negative (n=3<br>Failed (n=1<br><br>Fusion:<br>Negative (n=5, 100.0%)	**69/10**	Mutation:<br>TP53 (n=5 PIK3CA, ARID1A, RAD50, MET, NOTCH1 (n=1<br>Negative (n=3<br><br>CNV:<br>Negative (n=10<br><br>Fusion:<br>FGFR3 (n=1<br>Negative (n=9
**p-stage IIIa+IIIb**	**48/11**	**8/3**	Mutation:<br>TP53 (n=3, 100.0%)<br>FBXW7 (n=1, 33.3%)<br>NFE2L2 (n=1, 33.3%)<br><br>CNV:<br>Negative (n=3, 100.0%)<br><br>Fusion:<br>Negative (n=3, 100.0%)	**13/3**	Mutation:<br>TP53 (n=3, 100.0%)<br>SLX4 (n=1, 33.3%)<br><br>CNV:<br>Negative (n=3, 100.0%)<br><br>Fusion:<br>Negative (n=3, 100.0%)	**27/5**	Mutation:<br>TP53 (n=3, 60.0%)<br>NFE2L2, PTEN, FANCD2 (n=1, 20.0%)<br>Negative (n=1, 20.0%)<br>Failed (n=1)<br><br>CNV:<br>Negative (n=4, 100.0%)<br>Failed (n=1)<br><br>Fusion:<br>Negative (n=4, 100.0%)<br>Failed (n=1)
**Missing**	**2**					**2**	

Table A10b. NGS testing primary tumour, stratified by p-stage and later metastasis status: Cases that did not develop metastasis, cases that developed brain metastasis and cases that developed extracranial metastasis.
LUAD: Lung adenocarcinoma LUSC: Lung squamous cell carcinoma

Table A11. Mutations. Time to metastasis and time from metastasis to death in LUAD and LUSC. KRAS and EGFR excluded.

	Time to met. (years)***	HR*/ Chi²**	Time to BM (years)***	HR*/ Chi²**	Time from BM to death (years)***	HR*/ Chi²**	Time to ECM (years)***	HR*/ Chi²**	Time from ECM to death (years)***	HR*/ Chi²**
**LUAD**										
**TP53 (WT ref.)**	**0.82**	**2.06, 0.97-4.37, p=0.059/ 3.72, 0.054**	**0.74**	**n.s./n.s.**	**2.96**	**0.08, 0.01-0.67, p=0.020/ 8.82, p=0.003**	**2.83**	**n.s.**	**0.86**	**n.s.**
**STK11 (WT ref.)**	**0.58**	**3.57, 0.82-15.59, p=0.090/ 3.28, 0.070**	-	-	-	-	**0.58**	**14.54, 2.63-80.27, p=0.002/ 16.52, p<0.001**	**0.68**	**n.s.**
**CREBBP (WT ref.)**	**0.55**	**8.60, 1.82-40.76, p=0.007/ 10.66, 0.001**	**0.62**	***7.56, 0.88-65.38, p=0.066/4.70, p=0.030 OBS***	**2.11**	**n.s./n.s.**	**0.47**	***10.03, 1.04-96.46, p=0.046/ 6.10, p=0.014 OBS***	**0.44**	**n.s.**
**MAP2K1 (WT ref.)**	**0.75**	**5.88, 1.30-26.63, p=0.021/ 6.82, 0.009**	**0.75**	**9.59, 1.98-46.51, p=0.005/ 11.85, p<0.001**	**0.90**	**n.s./n.s.**	-	-	-	-
**BRCA2 (WT ref.)**	**1.58**	**3.27, 0.76-14.07, p=0.112/ 2.84, p=0.092**	**1.58**	**5.09, 1.14-22.81, p=0.033/ 5.61, 0.018**	**2.27**	**n.s./ n.s.**	-	-	-	-
STAT3 (WT ref.)	0.44	12.85, 1.50-110.01, 0.020/ 9.10, 0.003	-	-/n.s.	-	-	0.44	*62.97, 3.94-1007.06, p=0.003/ 30.48, p<0.001 OBS*	0.82	n.s.
CBL (WT ref.)	*0.75*	*5.49, 0.71-42.53, p=0.103/3.36, p=0.067 CBL*	*0.75*	*8.57, 1.05-69.73, p=0.045/ 5.85, p=0.016 OBS*	*2.68*	*n.s./ n.s.*	-	-	-	-

NF1 (WT ref.)	*0.77*	*4.98, 0.65-38.31, p=0.123/ 2.93, p=0.087 OBS*	*0.77*	*7.39, 0.92-59.16, p=0.059/ 4.91, p=0.027 OBS*	-	*-/ n.s.*	-	-	-	-
U2AF1 (WT ref.)	*0.29*	*21.90, 2.28-210.60, p=0.008/ 14.96, p<0.001 OBS*	*0.29*	*32.98, 2.99-363.77, p=0.004/ 20.66, p<0.001 OBS*	*2.6*	*n.s./ n.s.*	-	-	-	-
PTCH1 (WT ref.)	*0.36*	*n.s./n.s. OBS*	*0.36*	*7.33, 0.80-66.79, p=0.077/ 4.29, p=0.038 OBS*	*6.68*	*-/n.s.*	-	-	-	-
BRCA1 (WT ref.)	*1.44*	*n.s./n.s. OBS*	*1.44*	*5.10, 0.66-39.55, p=0.119/ 3.02, p=0.082 OBS*	*2.10*	*n.s./ n.s.*	-	-	-	-
RAD50 (WT ref)	*2.26*	*n.s./n.s.*	-	-	-	-	*2.26*	*7.39, 0.91-60.29, p=0.062/ 4.82, p=0.029 OBS*	*0.90*	-
ATRX (WT ref)	*0.73*	*n.s./n.s. OBS*	*0.73*	*n.s./n.s. OBS*	*0.90*	*n.s./ 3.41, p=0.065*	-	*n.s./n.s.*	-	-
**LUSC**										
**TP53** *(WT ref)*	**1.73**	**n.s./n.s.**	**2.32**	**n.s.**	**0.36**	**n.s./n.s.**	**1.38**	**n.s./n.s.**	**1.38**	**n.s./n.s.**
ATRX (WT ref)	*0.75*	*6.55, 0.79-54.43, p=0.082/ 4.03, 0.045*	-	-	-	-	*0.75*	*7.81, 0.91-66.86, p=0.061/ 4.94, p=0.026 OBS*	*3.33*	*n.s./n.s.*
**NFE2L2** *(WT ref)*	**0.44**	**5.07, 1.63-15.80, p=0.005/ 9.67, 0.002**	**0.35**	*6.65, 0.66-67.58, p=0.109/ 3.38, p=0.066 OBS*	**0.27**	**-**	**0.52**	**4.69, 1.27-17.26, p=0.020/ 6.53, p=0.011**	**0.41**	**n.s./n.s.**
CDKN2A (WT ref)	*0.75*	*6.55, 0.79-54.43, p=0.082/ 4.03, 0.045 OBS*	-	-	-	-	*0.75*	*7.81, 0.91-66.86, p=0.061/ 4.94, p=0.026 OBS*	*3.33*	*n.s./n.s.*

SLX4 (WT ref)	*0.73*	*7.98, 0.93-68.34, p=0.058/ 5.09, 0.024 OBS*	-	-	-	-	*0.73*	*9.92, 1.11-88.83, p=0.040/ 6.42, p=0.011 OBS*	*1.37*	*n.s./n.s.*
**FBXW7** *(WT ref)*	**1.03**	**n.s./ n.s.**	**0.52**	7.37, 0.75-72.74, p=0.087/ 4.01, p=0.045 OBS	0.43	**-**	***1.73***	*n.s./n.s. OBS*	**-**	**n.s./n.s.**
**PIK3CA** *(WT ref)*	**0.66**	**n.s./ n.s.**	**-**	**-**	**-**	**-**	**0.66**	**3.56, 0.78-16.21, p=0.101/ 3.07, p=0.080**	**0.62**	**n.s./n.s.**

Table A11. LUAD: Lung adenocarcinoma. LUSC: Lung squamous cell carcinoma. ***Median time to event among patients who experienced the event during follow-up. * Cox regression. HR, 95% CI, p-value ** Log Rank Chi2, Pr>chi2 OBS: Only 1 case. Bold: More than 1 case. *Italics: Only 1 case*

Table A12a. EGFR PCR or NGS adenocarcinoma

	**EGFR POSITIVE**	**EGFR NEGATIVE**	**OR, 95% CI, p-value (logistic regression)**
**Number**	60 (100%)	444 (100%)	
**Sex**			
**Female**	43 (71.7%)	243 (54.7%)	ref
**Male**	17 (28.3%)	201 (45.3%)	0.48, 0.26-0.86, 0.014
**Age median**	68.9	67.3	n.s.
**Age </50 years**			
**<50 years**	5	16	Ref.
**>50 years**	55	428	0.41, 0.14-1.17, 0.095
**Smoking**			
**Never smoker**	28 (46.7%)	30 (6.8%)	ref
**Former/current**	32 (53.3%)	414 (93.2%)	0.08, 0.04-0.16, p<0.001
			**HR, 95% CI, p-value (cox regression)**
**No metastasis**	34 (56.7%)	269 (60.6%)	ref
**Metastasis**	26 (43.3%)	175 (39.4%)	n.s.
**Brain metastasis**	10 (16.7%)	70 (15.8%)	n.s.

**Extra cranial metastasis**	16 (26.7%)	105 (23.7%)	n.s.			
	**EGFR POSITIVE**	**EGFR NEGATIVE**	**HR, 95% CI, p-value (Cox regression, EGFR negative ref.)**	**Chi2, Pr>chi2 (log rank, EGFR negative ref.)**	**EGFR subtypes. HR, p-value (Cox regression, EGFR negative ref.), median time (year)**	**EGFR subtypes. Chi2, Pr>chi2 (log rank, EGFR negative ref.), median time (year)**
**Median time (year)*** **						
**--to metastasis**	3.02	1.55	n.s.	n.s.	n.s.	n.s.
**--to BM**	1.34	1.03	n.s.	n.s.	Exon 21 (L861Q):7.02, 1.71-28.72, p=0.007, 0.59.	Exon 21 (L861Q): 10.23, p=0.0014, 0.59.
**--ECM**	3.34	2.13	n.s.	n.s.	n.s.	n.s.
**--from metastasis to lung cancer death**	1.95	0.77	n.s.*	n.s.	n.s.	n.s.
**--from BM to lung cancer death**	3.31	0.77	HR 0.39, 0.17-0.91, p=0.029	5.16, p=0.02	n.s.	n.s.
**--from ECM to lung cancer death**	1.41	0.77	n.s.	n.s.	n.s.	n.s.
**--from metastasis to all cause death**	1.96	0.77	n.s.*	n.s.	n.s.*	n.s.
**--from BM to all cause death**	3.32	0.74	HR 0.43, 0.20-0.94, p=0.035	4.71, p=0.030	Exon 21 (L858R): 0.45, 0.026-1.36, p=0.097[5], 6.68	Exon 21 (L858R): 2.87, p=0.090, 6.68
**--from ECM to all cause death**	1.41	0.79	n.s.	n.s.	n.s.	n.s.
**--to lung cancer related death**	5.06	2.63	n.s.*	n.s.	n.s.*	n.s.
**--to all cause death**	5.18	3.15	HR 0.65, 0.44-0.98, p=0.039*	4.33, p=0.037	Exon 19 deletion: 0.52, 0.25-1.04, p=0.066*, 5.61	Exon 19 deletion: 3.40, p=0.065, 5.61

Table A12a. Ref: Reference group. N.s.: Not significant, p>0.1 Cox: Cox regression. *Schoenfeld residuals test (χ^2, P>|χ^2|); P<0.05 indicates PH violation. ***Time to event among patients who experienced the event during follow-up.

Table A12b. EGFR PCR or NGS adenocarcinoma stratified by p-stage and later metastasis

	**Brain metastasis**			**Extracranial metastasis**			**No metastasis**		
	**Total**	**EGFR positive (n, % of total)**	**EGFR negative (n, % of total)**	**Total**	**EGFR positive (n, % of total)**	**EGFR negative (n, % of total)**	**Total**	**EGFR positive (n, % of total)**	**EGFR negative (n, % of total)**
**p-stage Ia+Ib**	30	3 (10.0%)	27 (90.0%)	53	9 (17.0%)	44 (83.0%)	205	25 (12.2%)	180 (87.5%)
**p-stage IIa+IIb**	26	4 (15.4%)	22 (84.6%)	43	5 (11.6%)	38 (88.4%)	69	6 (8.7%)	63 (91.3%)
**p-stage IIIa+IIIb**	24	3 (12.5%)	21 (87.5%)	25	2 (8.0%)	23 (92.0%)	29	3 (10.3%)	26 (89.7%)

Table A13. Subtypes EGFR mutations

EGFR mutation		Total	Metastasis	Brain metastasis	Extracranial metastasis	No metastasis
			Number	Number	Number	
**Total**		504 (100%)	201 (100%)	80 (100%)	111 (100%)	303 (100%)
**Negative**		444 (88.1%)	175 (87.1%)	70 (87.5%)	105 (86.8%)	269 (88.8%)
**Positive**		60 (11.9%)	26 (12.9%)	10 (12.5%)	16 (13.22%)	34 (11.2%)
**Total EGFR pos.**		60 (100%)	26 (100%)	10 (100%)	16 (100%)	34 (100%)
	Exon 18	4 (6.7%)	2 (7.7%)	1 (0.1%)	1 (6.3%)	2 (5.9%)
	Exon 20, non- T790M	7 (11.7%)	4 (15.4%)	1 (10.0%)	3 (18.8%)	3 (8.8%)
	Exon 21 L858R	23 (38.3%)	9 (34.6%)	3 (30.0%)	6 (37.5%)	14 (41.2%)
	Exon 21 L861Q	3 (5.0%)	2 (7.7%)	2 (20.0%)	-	1 (2.9%)
	Exon 19 del.	23 (38.3%)	9 (34.6%)	3 (30.0%)	6 (37.5%)	14 (41.2%)

Table A14a. KRAS PCR or NGS adenocarcinoma

	KRAS positive	KRAS negative	OR, 95% CI, p-value (logistic regression)
**Number**	120 (100%)	173 (100%)	
**Sex**			
**Female**	76 (63.3%)	97 (56.1%)	ref
**Male**	44 (36.7%)	43 (43.9%)	n.s.
**Age median**	66.8	66.6	n.s.

**Smoking**						
**Never smoker**	7 (5.8%)	35 (20.2%)	ref.			
**Former/current**	113 (94.2%)	138 (79.8%)	4.09, 1.75-9.57, p=0.001[1]			
			**HR, 95% CI, p-value (cox regression)**			
**No metastasis**	69 (57.5%)	110 (63.6%)	Ref.			
**Metastasis**	51 (42.5%)	63 (36.4%)	n.s.			
**Brain metastasis**	27 (22.5%)	22 (12.7%)	1.95, 1.11-3.45, p=0.019			
**Extra cranial metastasis**	24 (20.0%)	41 (23.7%)	n.s.			
	**KRAS positive**	**KRAS negative**	**HR, 95% CI, p-value (Cox regression, KRAS negative ref.)**	**KRAS. Chi2, Pr>chi2 (log rank, KRAS negative ref.)**	**KRAS subtypes. HR, 95% CI, p-value (Cox regression) KRAS negative ref., median time (year)[10]**	**KRAS subtypes. Chi2, Pr>chi2 (log rank) KRAS negative ref., median time (year)[10]**
**Median time (year)[10]**						
**--to metastasis**	1.44	1.97	n.s.	n.s.	(G12V): 2.04, 0.1.12-3.71, 0.019, 0.95	(G12V): 5.79, p=0.016, 0.95
**--to BM**	1.30	1.00	1.96, 1.12-3.45, p=0.019	5.73, 0.017	(G12C): 1.87, 0.94-3.72, p=0.073, 1.30<br>(G12V): 2.98, 1.27-6.98, p=0.012, 0.64<br>(G13D): 7.71, 1.03-57.45, p=0.046, 5.01<br>(Q61H): 4.61, 1.38-15.42, p=0.013, 1.50	(G12C): 3.31, p=0.069, 1.30<br>(G12V): 6.98, p=0.008, 0.64<br>(G13D): 5.85, 0.016, 5.01<br>(Q61H): 7.38, 0.007, 1.50
**--to ECM**	2.08	3.02	n.s.	n.s.	(G12R): 7.60, 1.03-56.21, p=0.047, 2.66<br>(G12S): 6.06, 0.82-45.02, p=0.078, 0.86	(G12R): 5.75, p=0.017, 2.66<br>(G12S): 3.98, p=0.046, 0.86
**--from metastasis to lung cancer death**	1.05	0.84	n.s.	n.s.	(G12S): 36.68, 3.79-355.22, p=0.002, 0.08	(G12S): 19.02, p<0.001, 0.08
**--from BM to lung cancer death**	0.87	1.41	1.72, 0.92-3.20, p=0.088	2.99, 0.084	(G12C): 1.97, p=0.077,0.93-4.15, 0.72	n.s.
**--from ECM to lung cancer death**	1.28	0.69	n.s.	n.s.	(G12S): 54.62, 3.40-877.34, p=0.005, 0.08	(G12S): 18.76, p<0.001, 0.08
**--from metastasis to all cause death**	1.05	0.84	n.s.	n.s.	(G12S): 36.61, 3.78-354.34, p=0.002, 0.08	(G12S): 19.02, p<0.001, 0.08
**--from BM to all cause death**	0.79	1.41	1.64, 0.91-2.96, p=0.102	2.73, 0.099	(G12C): 1.88, 0.92-3.84, p=0.084, 0.38	(G12C): 2.69, p=0.101, 0.38
**--from ECM to all cause death**	1.28	0.69	n.s.	n.s.	(G12S): 54.58, 3.40-876.34, p=0.005, 0.08	(G12S): 18.76, p<0.001, 0.08

**--to lung cancer related death**	2.89	3.24	1.44, 1.00-2.07, p=0.050	3.88, 0.049	(G12S): 5.42, 0.74-39.75, p=0.096, 0.93<br>(G12V): 1.79, 0.99-3.26, p=0.055, 3.63<br>(Q61H): 2.45, 0.99-6.11, 0.053, 4.99	(G12S): 4.10, p=0.043, 0.93<br>(G12V): 3.83, p=0.050, 3.63<br>(Q61H): 3.89, p=0.049, 4.99
**--to all cause death**	3.44	3.66	1.32, 0.98-1.76, p=0.073	3.23, 0.072	(G12S): 7.48, 1.81-30.87, p=0.005, 2.64	(G12S): 11.75, p<0.001, 2.64

Table A14. Ref: Reference group. N.s.: Not significant, p>0,1. Time to event among patients who experienced the event during follow-up.

Table A14b. KRAS PCR or NGS adenocarcinoma stratified by p-stage and later metastasis

	**Brain metastasis**			**Extracranial metastasis**			**No metastasis**		
	**Total**	**KRAS positive (n, % of total)**	**KRAS negative (n, % of total)**	**Total**	**KRAS positive (n, % of total)**	**KRAS negative (n, % of total)**	**Total**	**KRAS positive (n, % of total)**	**KRAS negative (n, % of total)**
**p-stage Ia+Ib**	18	12 (66.7%)	6 (33.3%)	31	12 (38.7%)	19 (61.3%)	127	52 (40.9%)	75 (59.1%)
**p-stage IIa+IIb**	21	10 (47.6%)	11 (52.4%)	20	7 (35.0%)	13 (65.0%)	32	7 (21.9%)	25 (78.1%)
**p-stage IIIa+IIIb**	10	5 (50.0%)	5 (50.0%)	14	5 (35.7%)	9 (64.3%)	20	10 (50.0%)	10 (50.0%)

Table A15. Subtypes KRAS mutations

**KRAS mutation**		**Metastasis**	**Brain**	**Extracranial metastasis**	**No metastasis**
**Total**		114 (100%)	49 (100%)	65 (100%)	179
**Negative**		63 (55.3%)	22 (44.9%)	41 (63.1%)	110 (61.4%)
**Positive**		51 (44.7%)	27 (55.1%)	34 (52.3%)	69 (38.5%)
	G12A	1	1	0	6
	G12C	23	13	10	35
	G12D	8	2	16	15
	G12R	1	0	1	0
	G12S	1	0	1	1

	G12V	13	7	6	9
	G13D	1	1	0	0
	Q61H	3	3	0	3

Figure A1. Kaplan-Meier survival estimates metastasis by risk of brain metastasis by clinicopathological parameters

a.

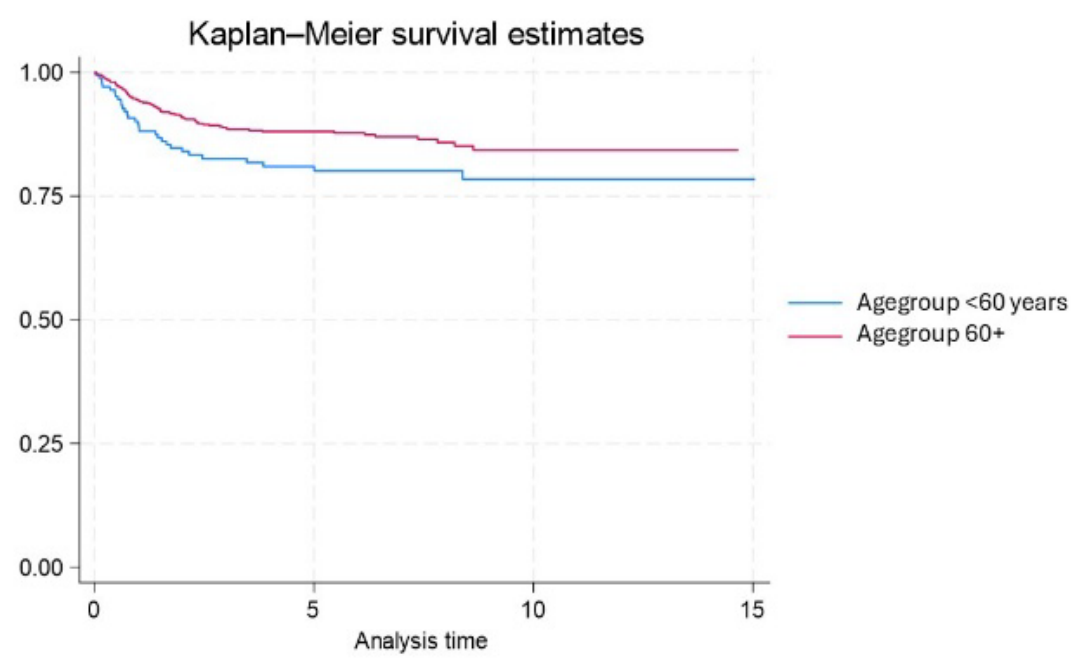

b.

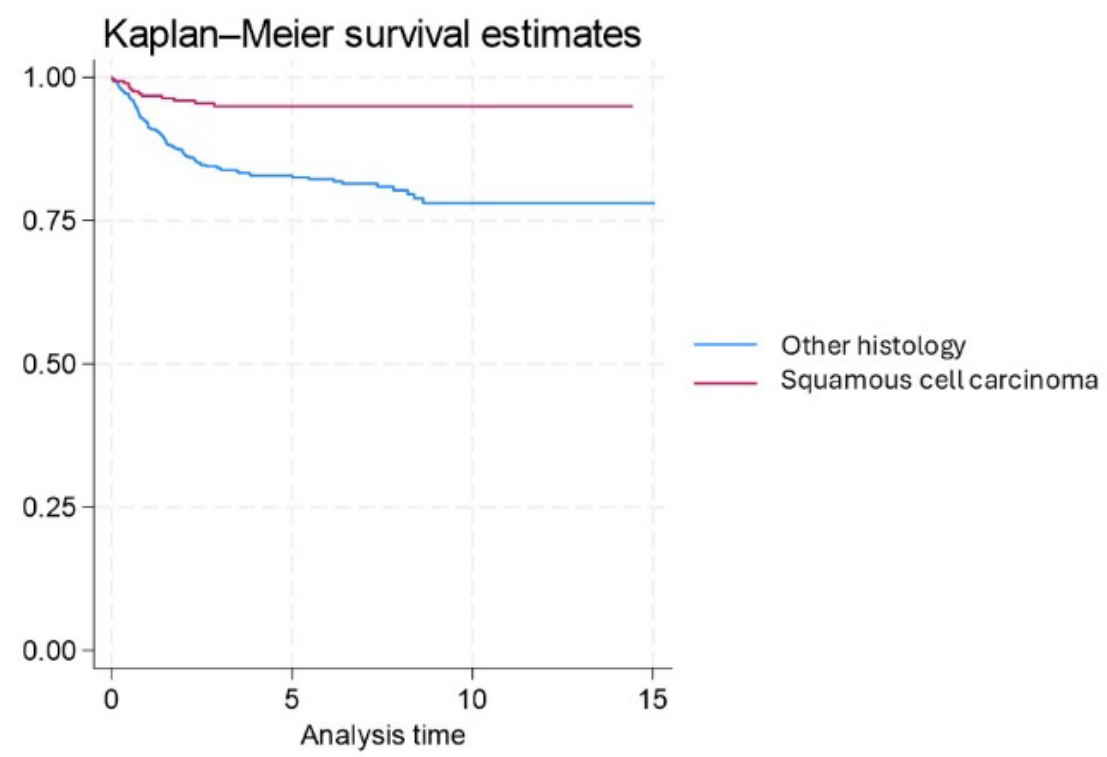

c.

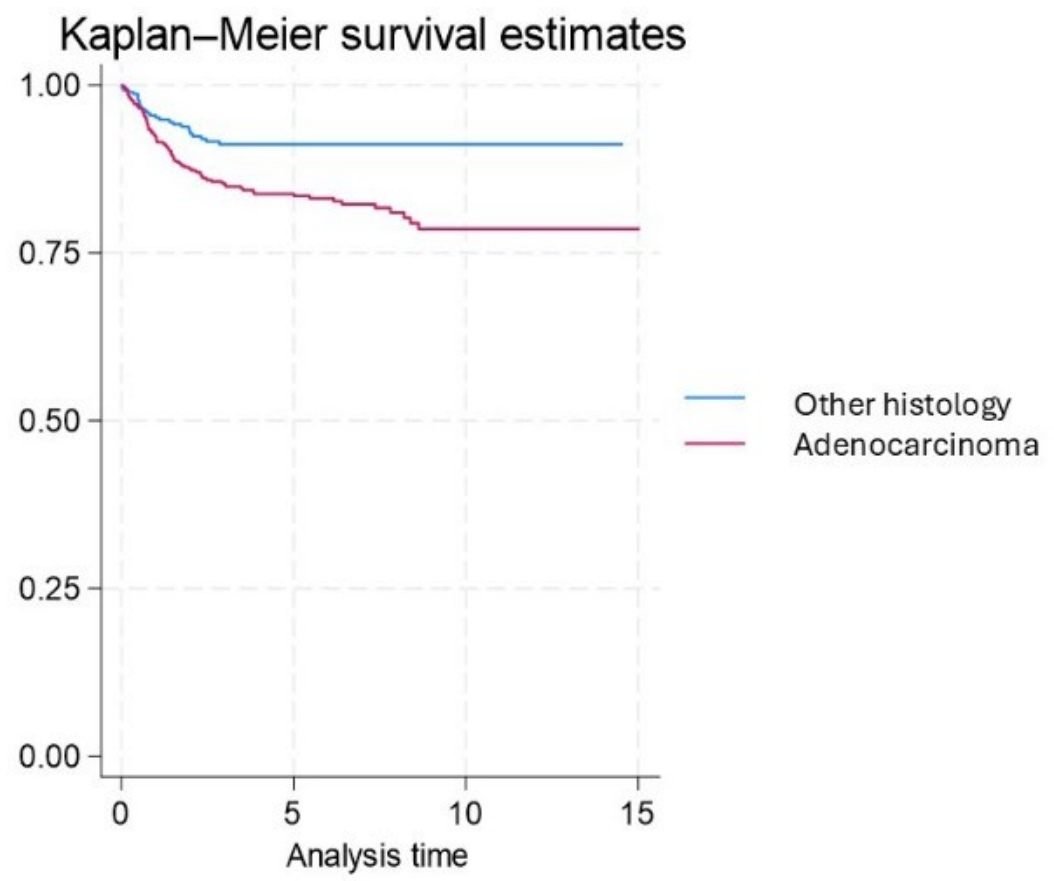

Kaplan–Meier survival estimates
1.00
0.75
0.50
0.25
0.00
0
5
10
15
Analysis time
Other histology
Adenocarcinoma

d.

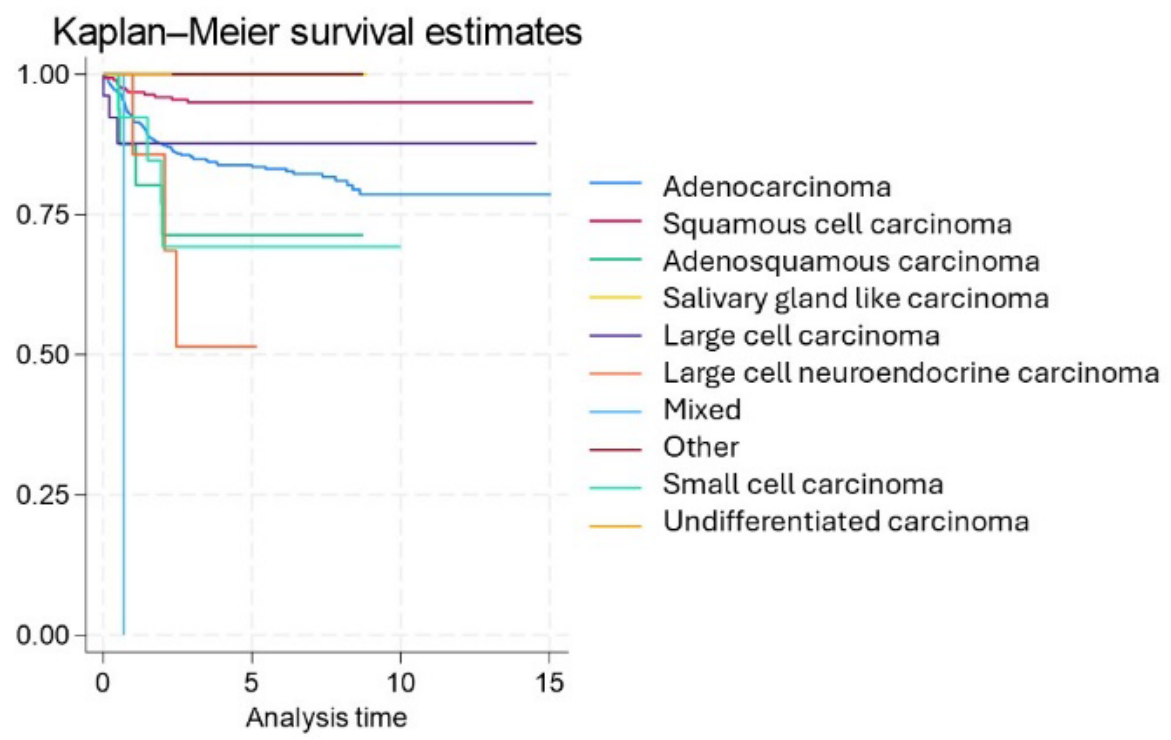

Kaplan–Meier survival estimates
1.00
0.75
0.50
0.25
0.00
0
5
10
15
Analysis time
Adenocarcinoma
Squamous cell carcinoma
Adenosquamous carcinoma
Salivary gland like carcinoma
Large cell carcinoma
Large cell neuroendocrine carcinoma
Mixed
Other
Small cell carcinoma
Undifferentiated carcinoma

e.

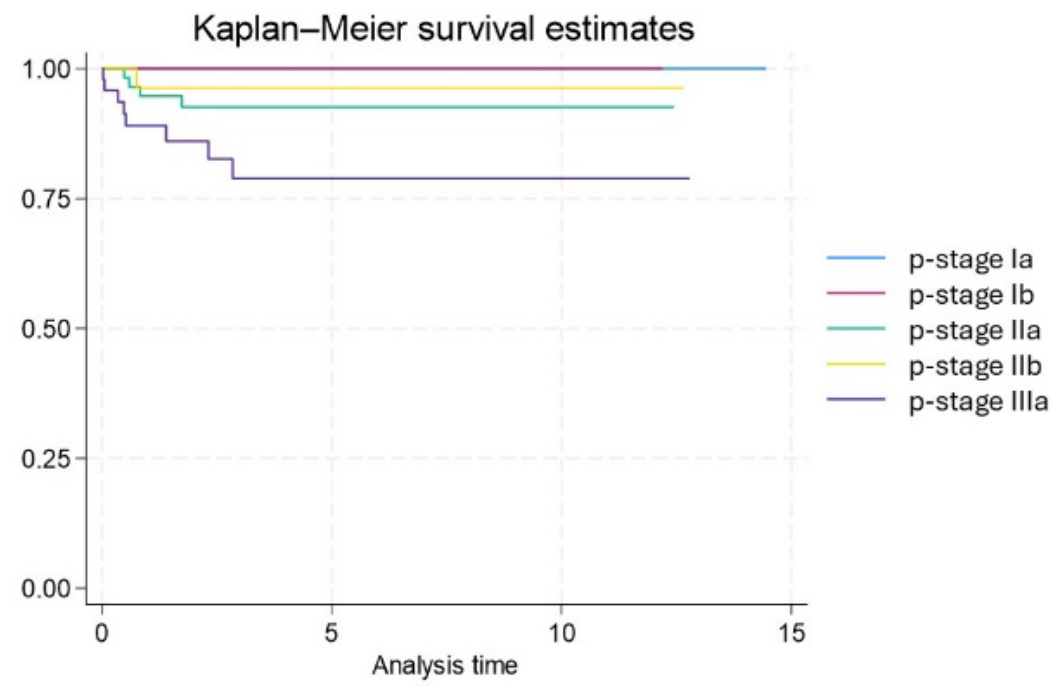

f.

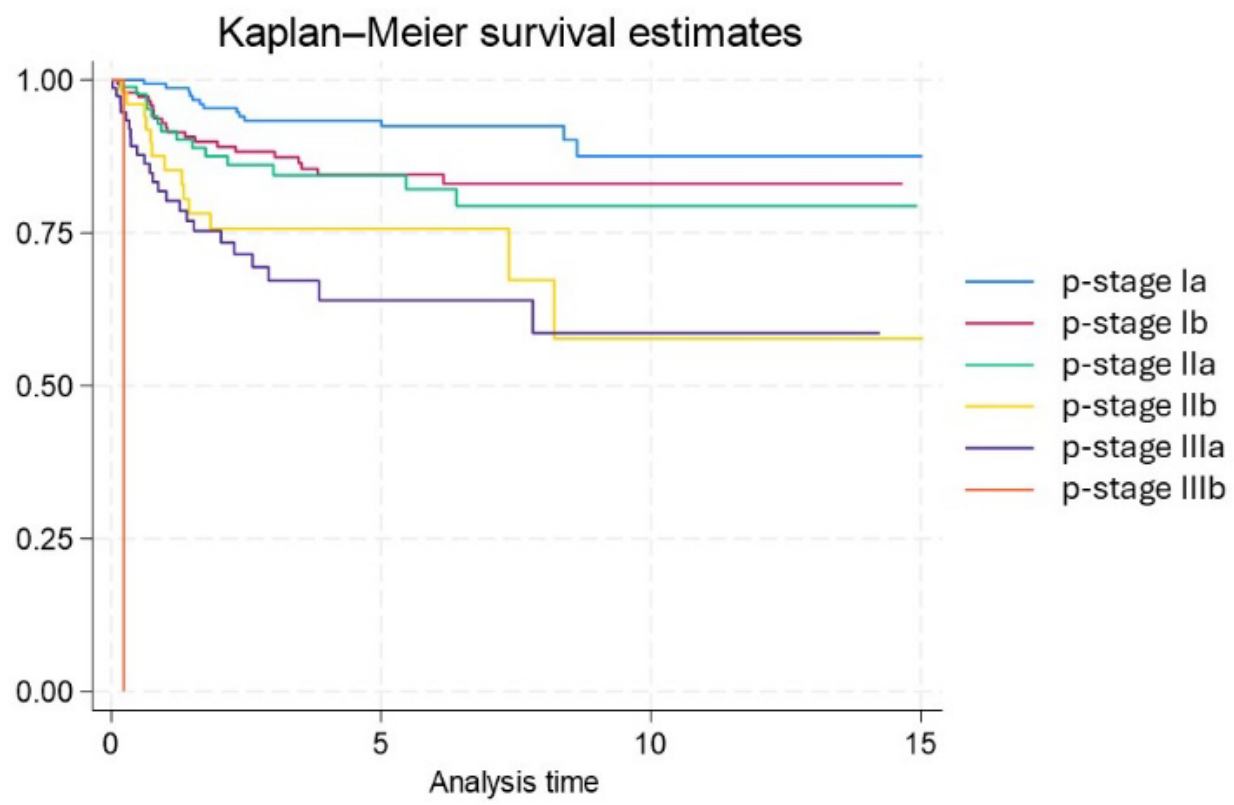

g.

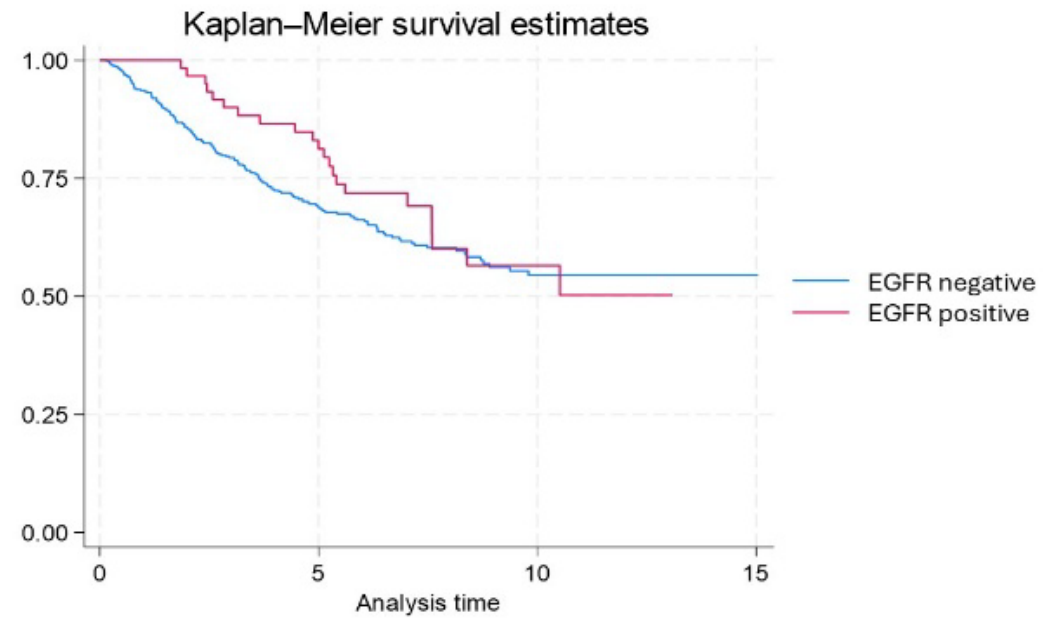

Figure A1. a. Estimated cumulative brain metastasis-free survival probability by year from surgery in agegroup <60 vs ≥ 60. b. Estimated cumulative brain metastasis-free survival probability by year from surgery in squamous cell carcinoma vs other histopathological subtypes c. Estimated cumulative brain metastasis-free survival probability by year from surgery in adenocarcinoma vs other histopathological subtypes d. Estimated cumulative brain metastasis-free survival probability by year from surgery in different histopathological subtypes e. Estimated cumulative metastasis-free survival probability by year from surgery in LUSC in different p-stage. f. Estimated cumulative metastasis-free survival probability by year from surgery in LUAD in different p-stage. g. Estimated cumulative survival probability by year from surgery to lung cancer specific death in EGFR positive versus negative

Figure A2. Kaplan-Meier survival estimates metastasis by risk of brain metastasis by molecular parameters

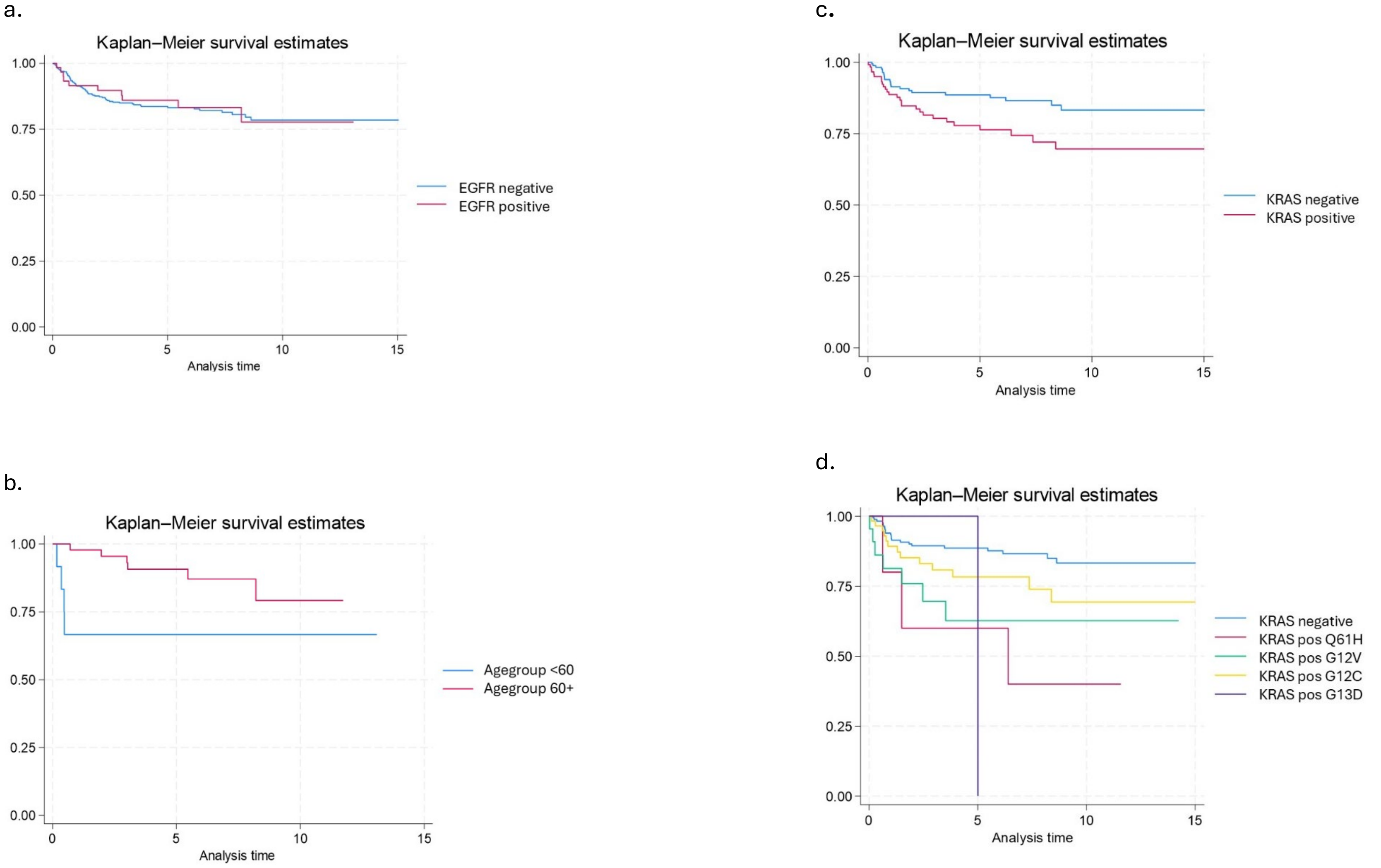

e.

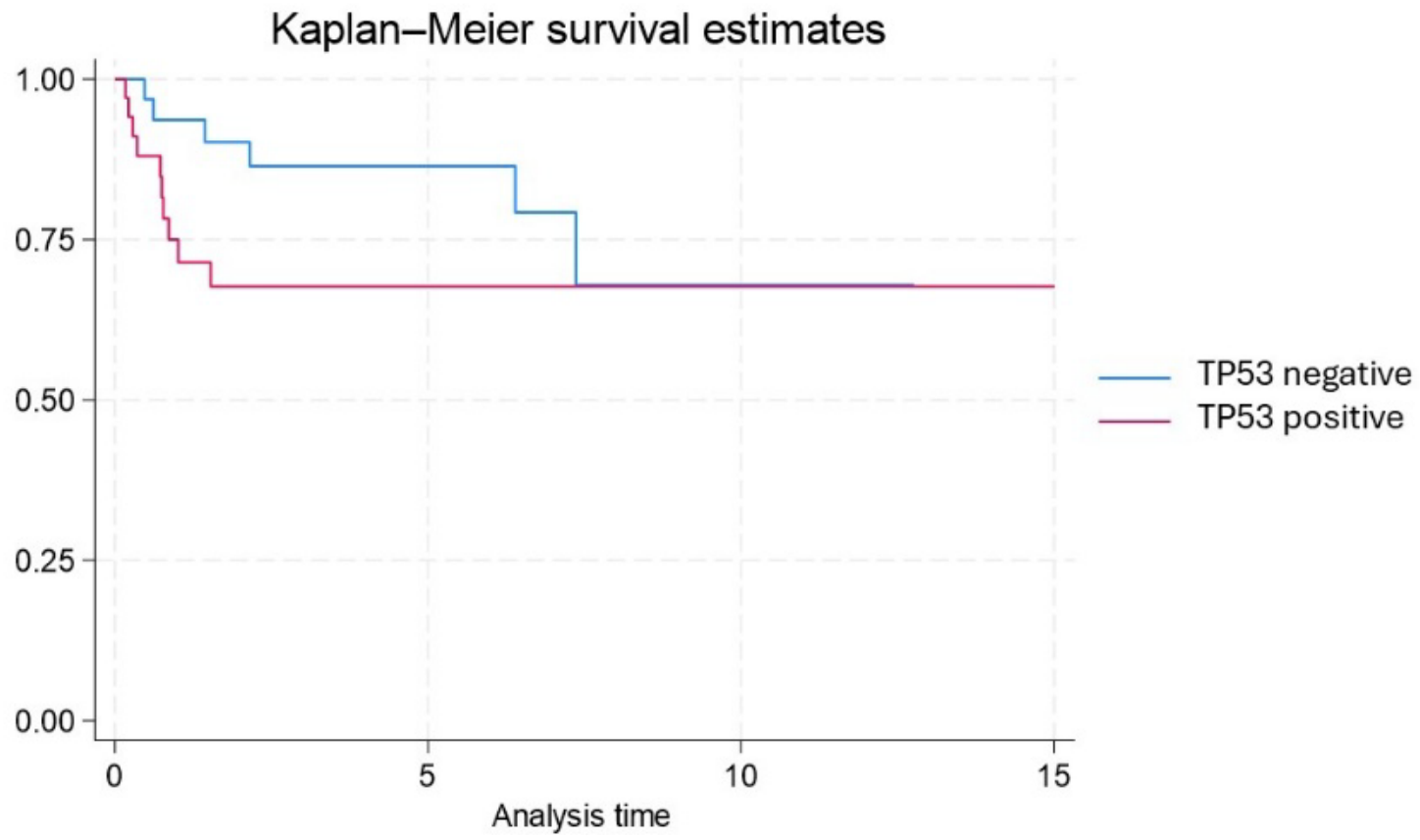

f.

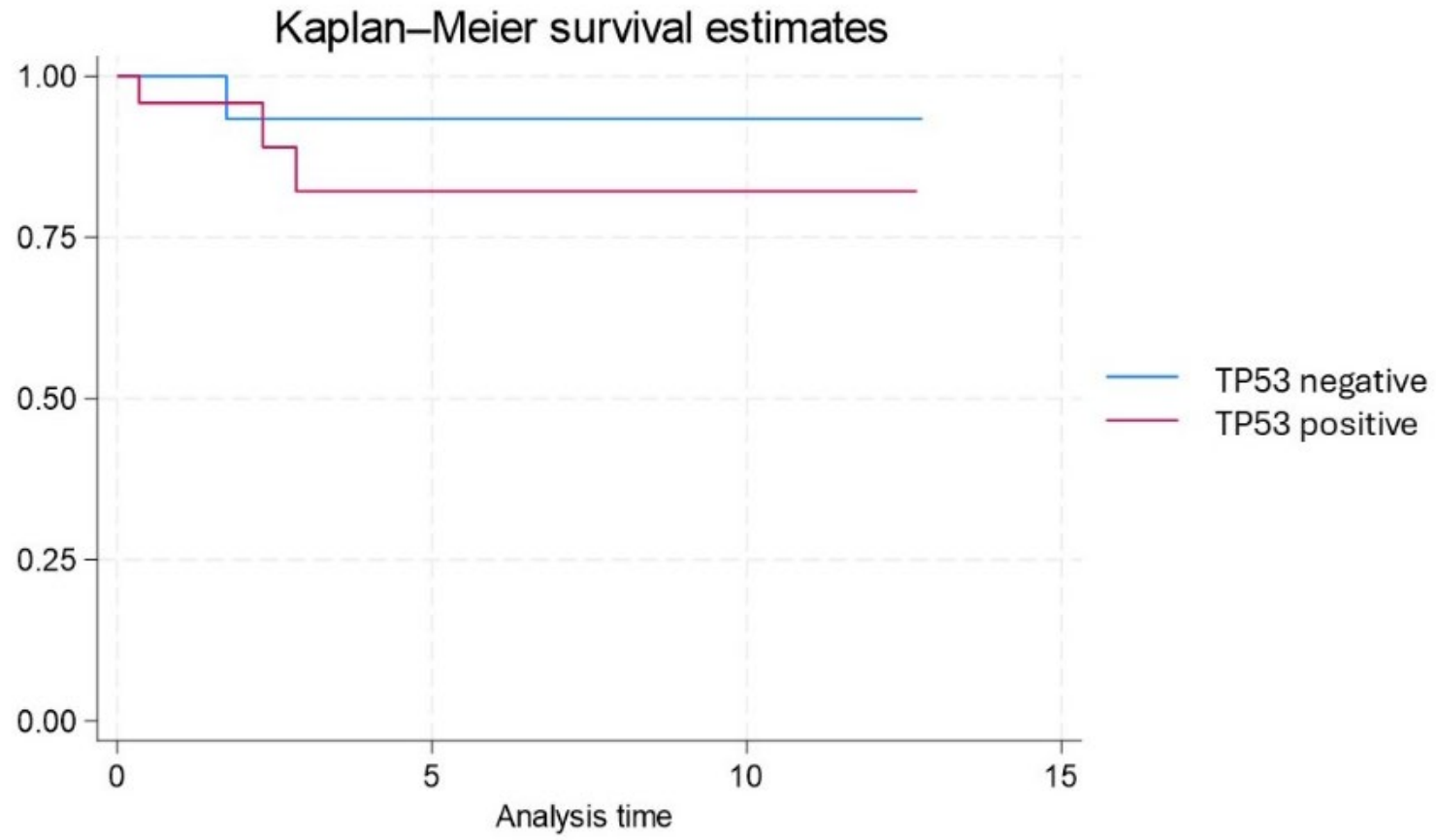

Figure A2. a. Estimated cumulative metastasis-free survival probability by year from surgery to metastasis in EGFR positive versus negative. b. Estimated cumulative brain metastasis-free survival probability by year from surgery to metastasis in agegroup <60 vs ≥ 60 in EGFR positive. c. Estimated cumulative brain metastasis-free survival probability by year from surgery to metastasis in KRAS positive versus negative. d. Estimated cumulative brain metastasis-free survival probability by year from surgery to metastasis in selected KRAS subtypes positive versus negative. e. Estimated cumulative brain metastasis-free survival probability by year from surgery to metastasis in LUAD in TP53 positive versus negative. f. Estimated cumulative brain metastasis-free survival probability by year from surgery to metastasis in LUSC in TP53 positive versus negative
